# Supplementary material for: Behavioral screening defines the molecular Parkinsonism-related subgroups in Drosophila
Source: Nat Commun. 2026 Mar 10;17:3761. doi: 10.1038/s41467-026-70303-8 (PMC13106710; doi:10.1038/s41467-026-70303-8)
Supplement: Supplementary file 1 — Supplementary Information [file 41467_2026_70303_MOESM1_ESM.pdf]

Supplementary figures

Supplementary Fig.1 for Fig.1

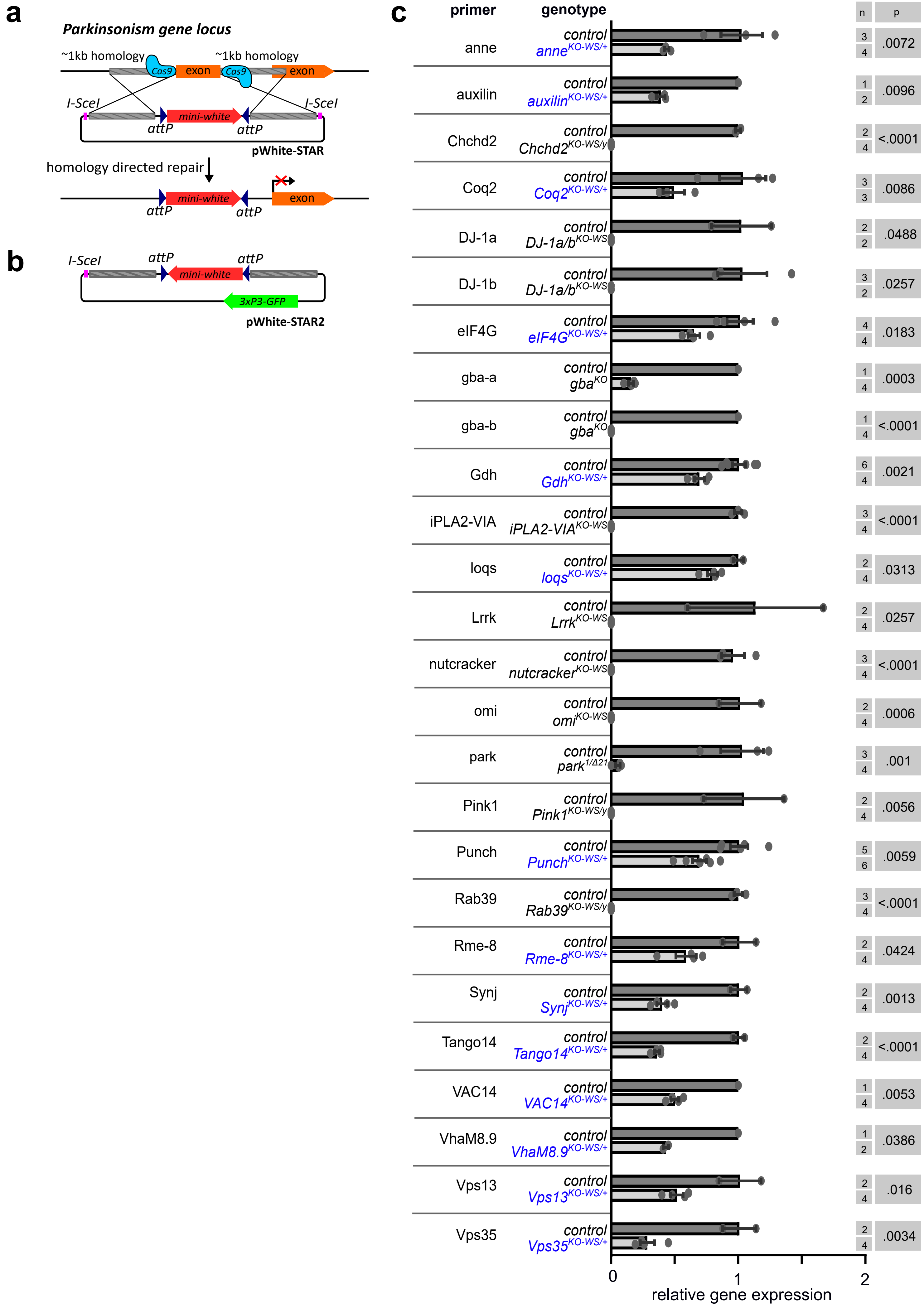

Supplementary Fig.1: Genetic strategy of Parkinsonism fly collection reduces gene expression levels, *related to Fig.1.* (a-b) Scheme of the genetic approach for the generation of the Parkinsonism mutant fly collection. The first common exon of all possible *Drosophila* transcripts of the targeted Parkinsonism gene was replaced by attP-flanked mini-*white* gene, by homology directed repair with the pWhite-STAR (a) or pWhite-STAR2 (b) using CRISPR/Cas9, creating a null mutant. *I-SceI* sites were utilized in the rare event of full donor plasmid integration (methods). For *park* we used $park^{1/\Delta 21}$. (c) Relative gene expression levels, measured by quantitative RT-PCR in young flies with primers to the indicated gene (left) in controls and Parkinsonism mutants. Data are expressed as a relative value compared to the endogenous *Drosophila* gene expression. The "/+" refers to heterozygosity (genotypes indicated in blue) and "/y" to hemizygosity. Unpaired t-test; Bars: mean ± SD; points are samples consisting of groups of animals, n and p values are indicated. Source data are provided as a Source Data file.

Supplementary Fig.2 for Fig1

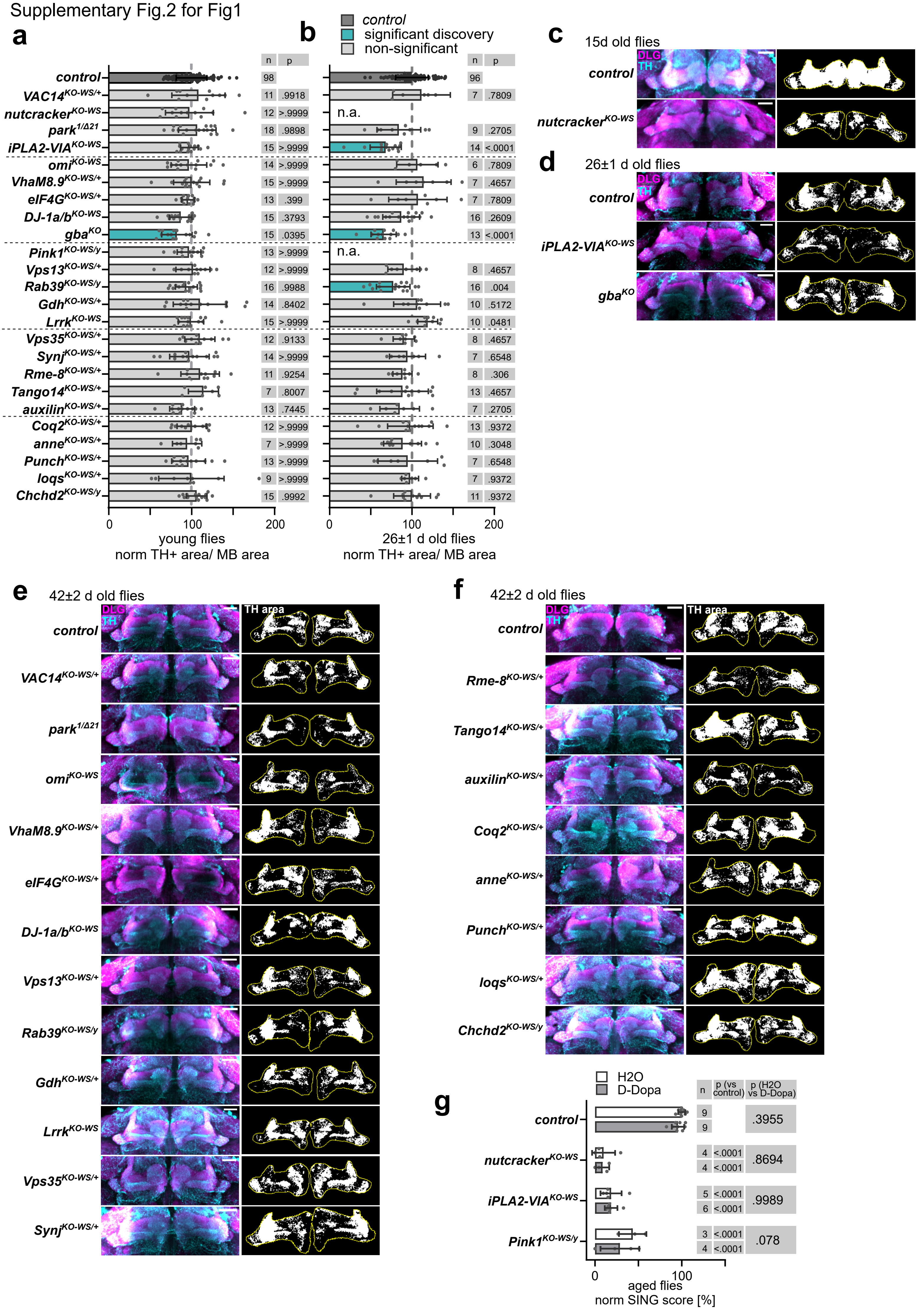

Supplementary Fig.2: Parkinsonism fly collection shows age-dependent dopaminergic defects, *related to Fig.1.* (a,b) Quantification of dopaminergic synaptic area within MB area at (a) 5±1 d and (b) 26±1 d after eclosion in mutants relative to the control. Due to their shorter lifespan *nutcracker*$^{KO\text{-}WS}$ were tested at 15 d, *Pink1*$^{KO\text{-}WS}$ at 22±2 d and are not included in (b). (a) One-way ANOVA with Dunnett's test and (b) ANOVA Kruskal-Wallis with Benjamini-Hochberg. Bars: mean ± SD; points are individual animals; n and p values are indicated. (c-f) Maximum projection confocal images of control and mutant fly brains at (c) 15±1 d, (d) 26±1 d and (e-f) 42±2 d after eclosion stained with anti-TH (cyan) and anti-DLG (magenta) antibodies, where DLG marks the post-synaptic site of MB. The black and white images represent the thresholded TH area (in white) of "middle z-plane" within the ROI (yellow, outline of MB). Scale bar: 20 µm. Representative images of quantification in Fig.1c, n indicated in graph. (g) SING quantification of aged flies treated with solvent control H2O or D-Dopa 10 d prior to the assay relative to H_2O treated control. Bars: mean ± SD; points represent groups of animals. Two-way ANOVA with Tukey's multiple comparison, n and p values are indicated. Source data are provided as a Source Data file. DLG, Discs-large; MB, mushroom body; SING, startle-induced negative geotaxis; TH, tyrosine hydroxylase.

Supplementary Fig.3 for Fig.1

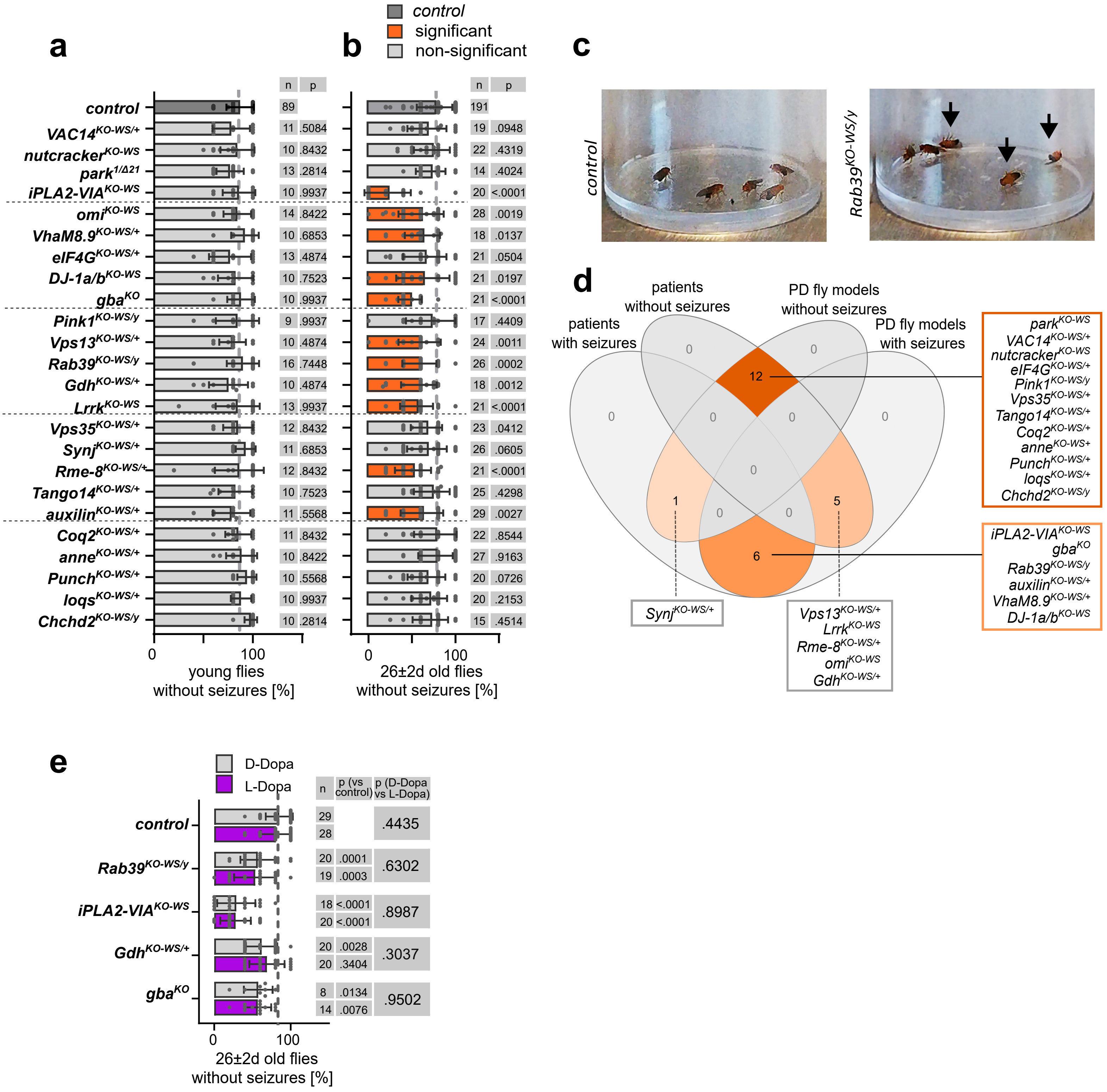

Supplementary Fig.3: Several Parkinsonism mutants show seizure-like behavior, *related to Fig.1.* (a,b) Quantification of Parkinsonism mutants without seizure-like behavior after sensory stimulation (vortex) at (a) 6±1 d (young) and (b) 26 ±2 d after eclosion (except for *nutcracker*$^{KO\text{-}WS}$ at 15±1 d and, *Pink1*$^{KO\text{-}WS}$ at 22±2 d). Orange colored bars represent $p \leq 0.01$, ANOVA Kruskal-Wallis with Benjamini-Hochberg compared. Bars: mean ± SD; points are groups of animals and $N \geq 9$. (c) Representative images of 25±1 d old control and *Rab39*$^{KO\text{-}WS}$ after seizure induction, black arrows indicate flies with seizure-like phenotypes. (d) Venn diagram representing the convergence of patients with familial forms of Parkinsonism described to suffer from seizures and fly mutants with or without seizure-like behavior. (e) Quantification of a subset of Parkinsonism mutants without seizure-like behavior after sensory stimulation (vortex) at 26 ±2 d after eclosion treated with L-Dopa or D-Dopa (control, grey bars) 10 d prior to the assay. Two-way ANOVA with Tukey's multiple comparison, n and p values are indicated; points are groups of animals. Bars: mean ± SD. Source data are provided as a Source Data file.

Supplementary Fig.4 for Fig.2

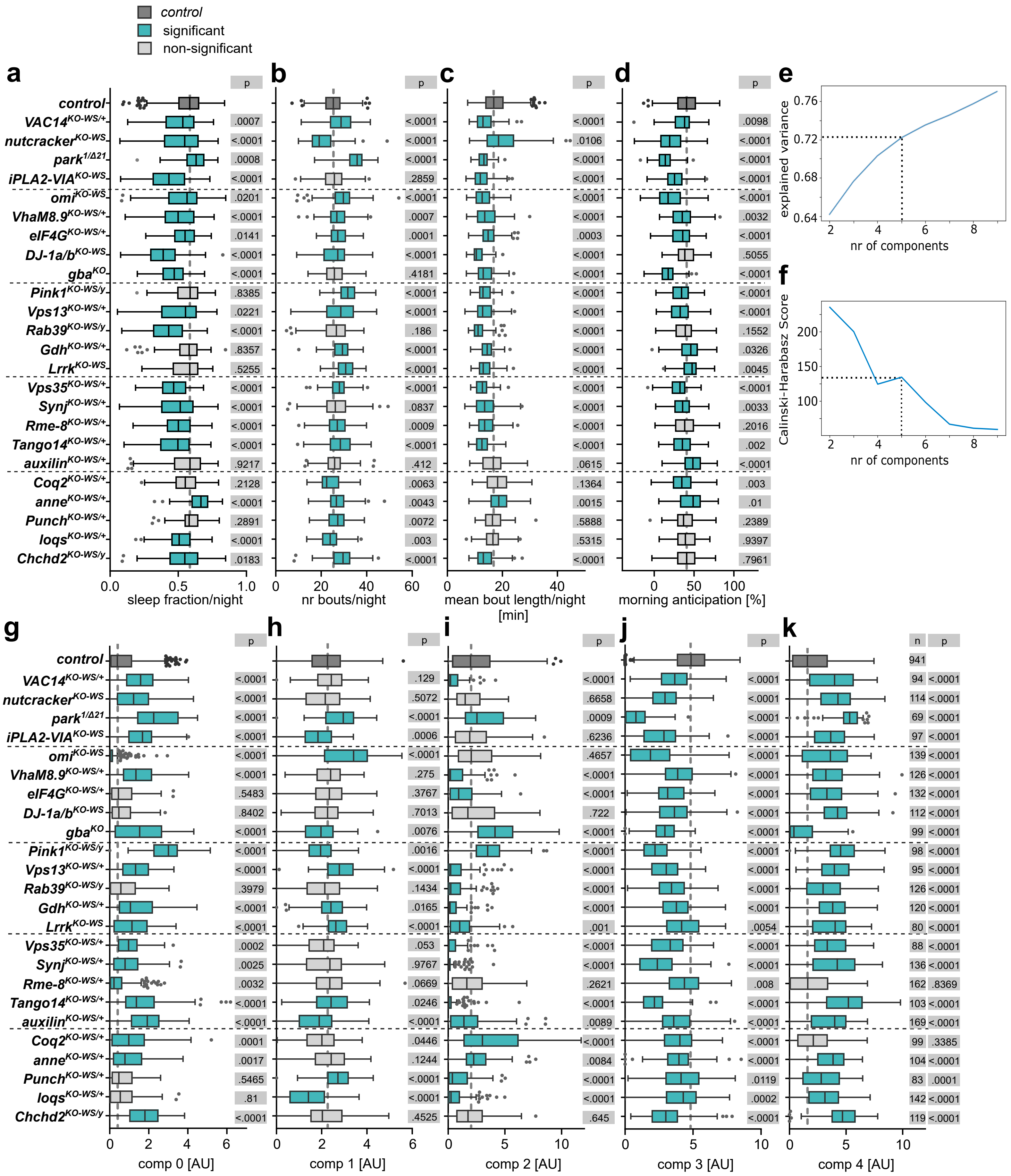

Supplementary Fig.4: Young Parkinsonism mutant flies display diverse behavioral defects, *related to Fig.2*. Quantification of (a) sleep fraction per night, (b) number of bouts per night, (c) mean bout length per night in min and (d) morning anticipation in % in Parkinsonism mutants averaged over 5 d. (e) Elbow method supports 5 components as meaningful number of features explaining 72 % of the variance. (f) The Calinski-Harabasz Index for optimal number of clusters displays a peak at 5 components indicating that 5 clusters provide a reasonable balance between more fine-grained clusters and cluster quality. (g-k) Quantification of components 0-4 represented by single flies in individual genotypes. (a-d, g-k) Box and whisker plot (Tukey method): median and IQR; statistical significance: ANOVA Kruskal-Wallis with Benjamini-Hochberg; from individual animals, p values are indicated, n values are the same for a-d and g-k and are indicated in k. Source data are provided as a Source Data file.

Supplementary Fig.5 for Fig.2

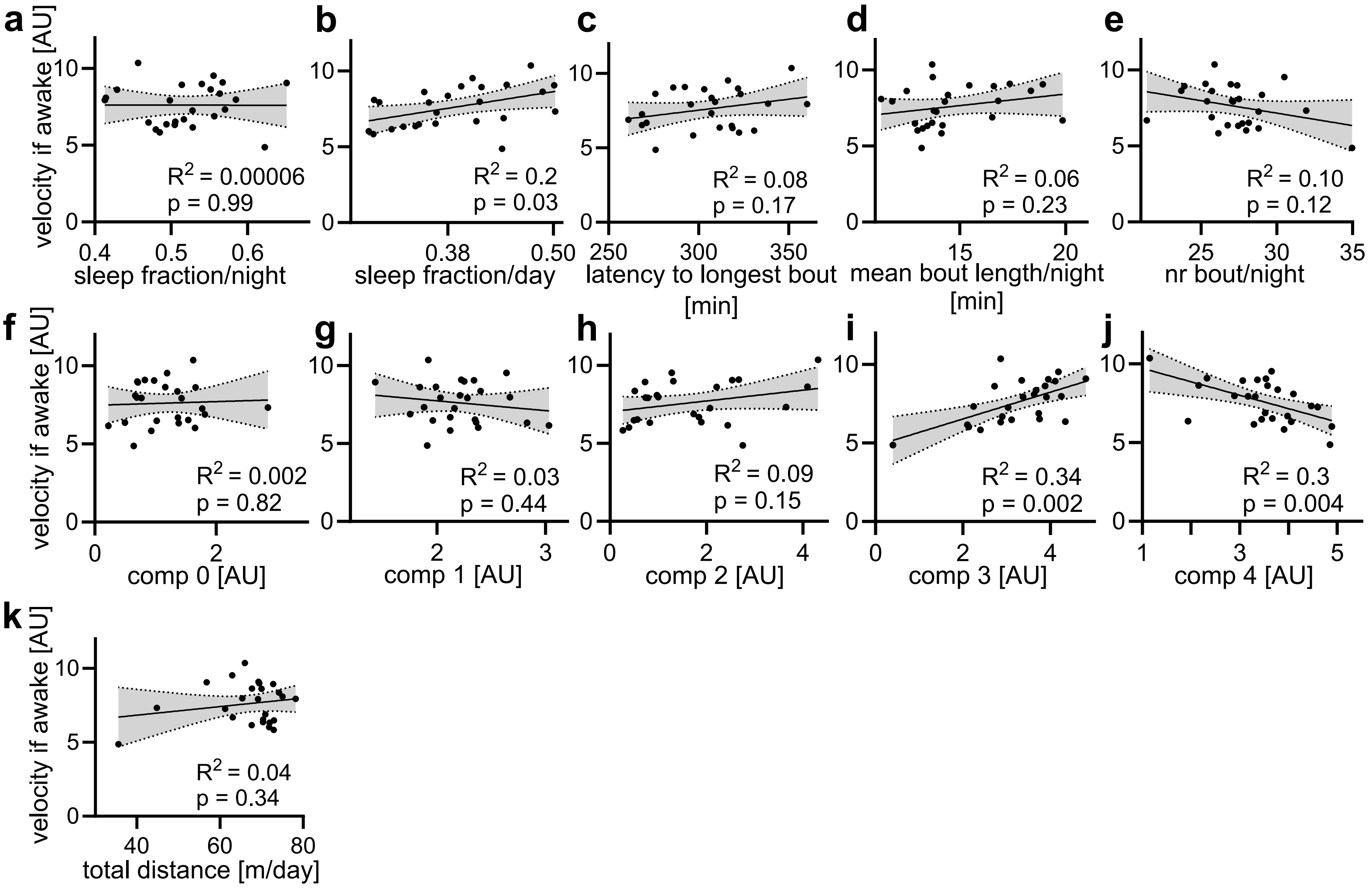

Supplementary Fig.5: Basic motor performance does not generally correlate with behavioral parameters or NMF components 0-4, *related to Fig.2*. Linear regression plots with 95% confidence intervals (grey area) of behavior parameters with velocity if awake [AU] (a-k). Dots represent the mean of Parkinsonism mutants. R^2 and p-values are indicated. Source data are provided as a Source Data file.

Supplementary Fig.6 for Fig.2

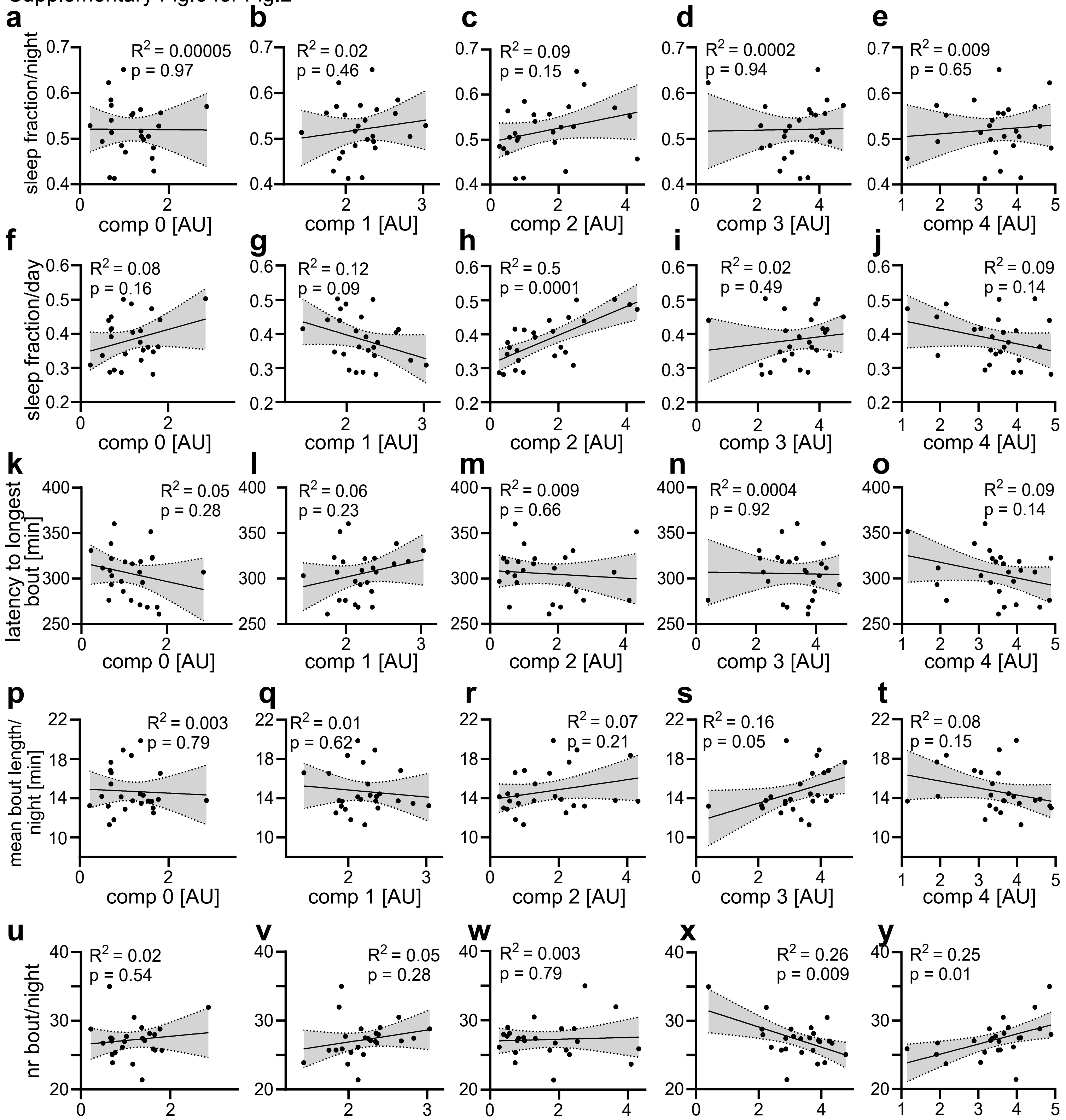

Supplementary Fig 6: Behavior features do not generally correlate with NMF parameters, *related to Fig.2*. (a-y) Linear regression plots with 95% confidence intervals (grey area) of behavioral features with NMF components. Dots represent the mean of Parkinsonism mutants. R2 and p-values are indicated. Source data are provided as a Source Data file.

Supplementary Fig.7 for Fig.2

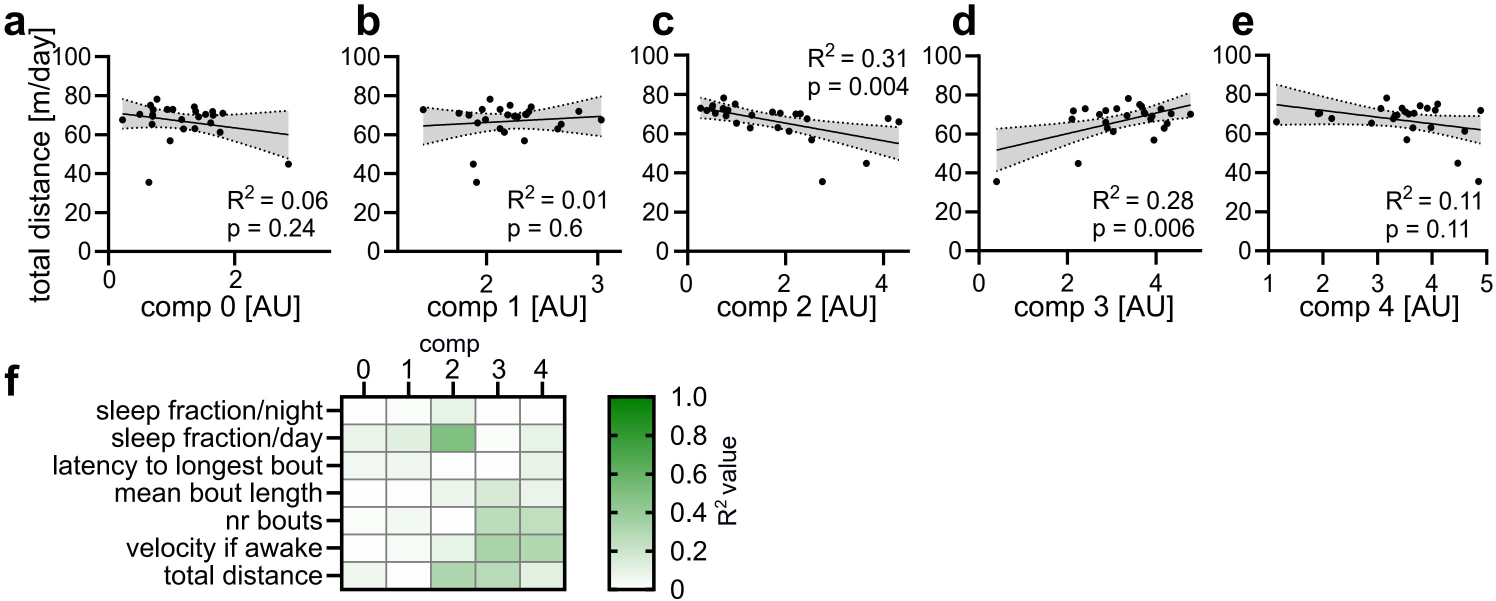

Supplementary Fig 7: Behavior features (total distance) do not generally correlate with NMF parameters, *related to Fig.2*. (a-e) Linear regression plots with 95% confidence intervals (grey area) of behavioral features with NMF components. Dots represent the mean of Parkinsonism mutants. R2 and p-values are indicated. (f) Summary heatmap of R^2 values of every behavioral parameter with component 0-4. Source data are provided as a Source Data file.

Supplementary Fig.8 for Fig.2

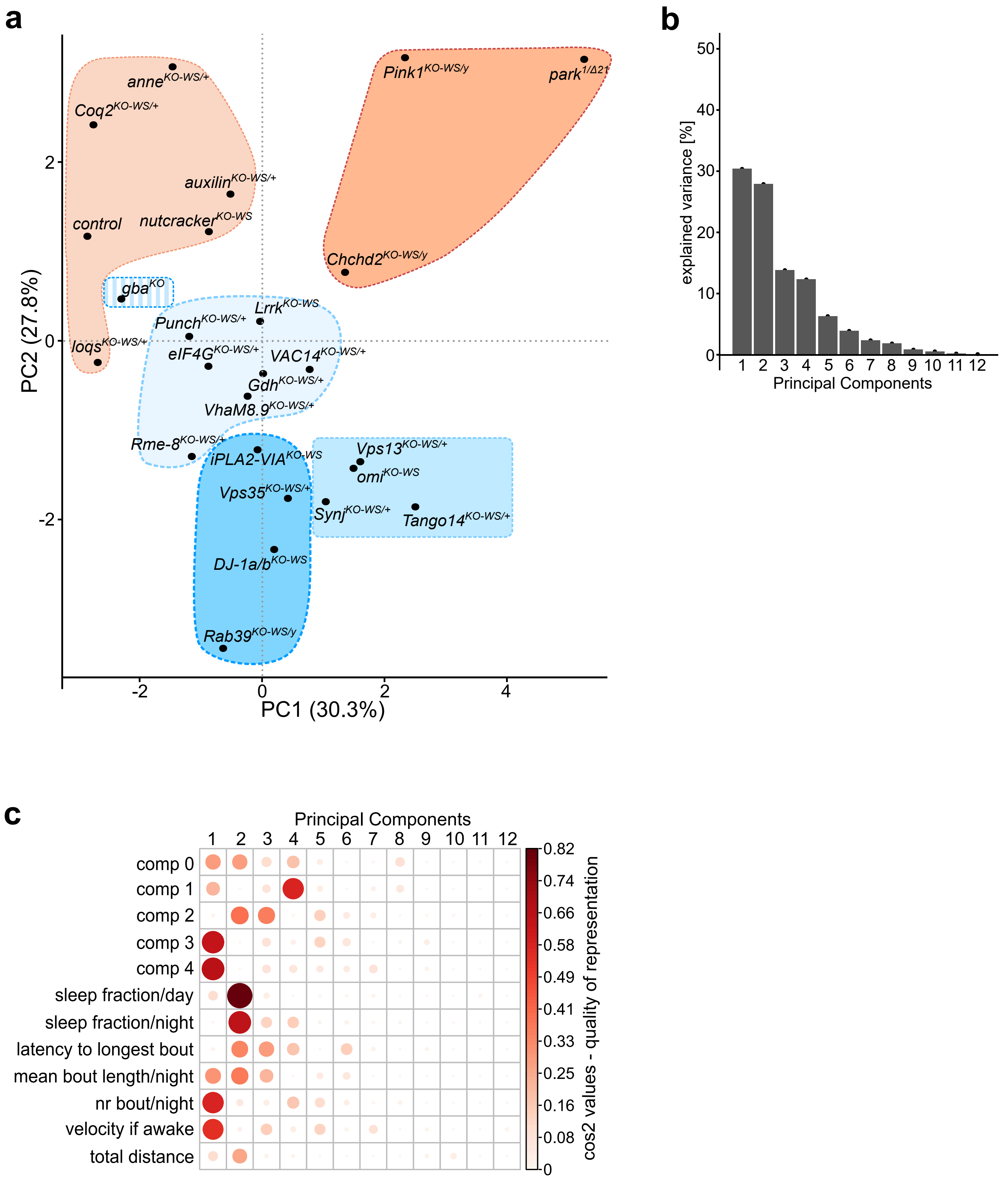

Supplementary Fig.8: Principal component analysis (PCA) supports the assignment of genes to groups identified with hierarchical clustering, *related to Fig.2*. (a) PCA plot of Parkinsonism mutants based on their scaled behavior features and boxes are inserted manually highlighting behavior groups, color coded as in Fig.2e. Principal component (PC) 1 and 2 explain 58% of total variance. (b) Screen plot describing the explained variance of individual PCs. (c) Cos2 plot describing the quality of representation of behavior parameters within individual PCs. Both dot size and color indicate the cos2 value. Comp, component; nr, number.

Supplementary Fig.9 for Fig.2

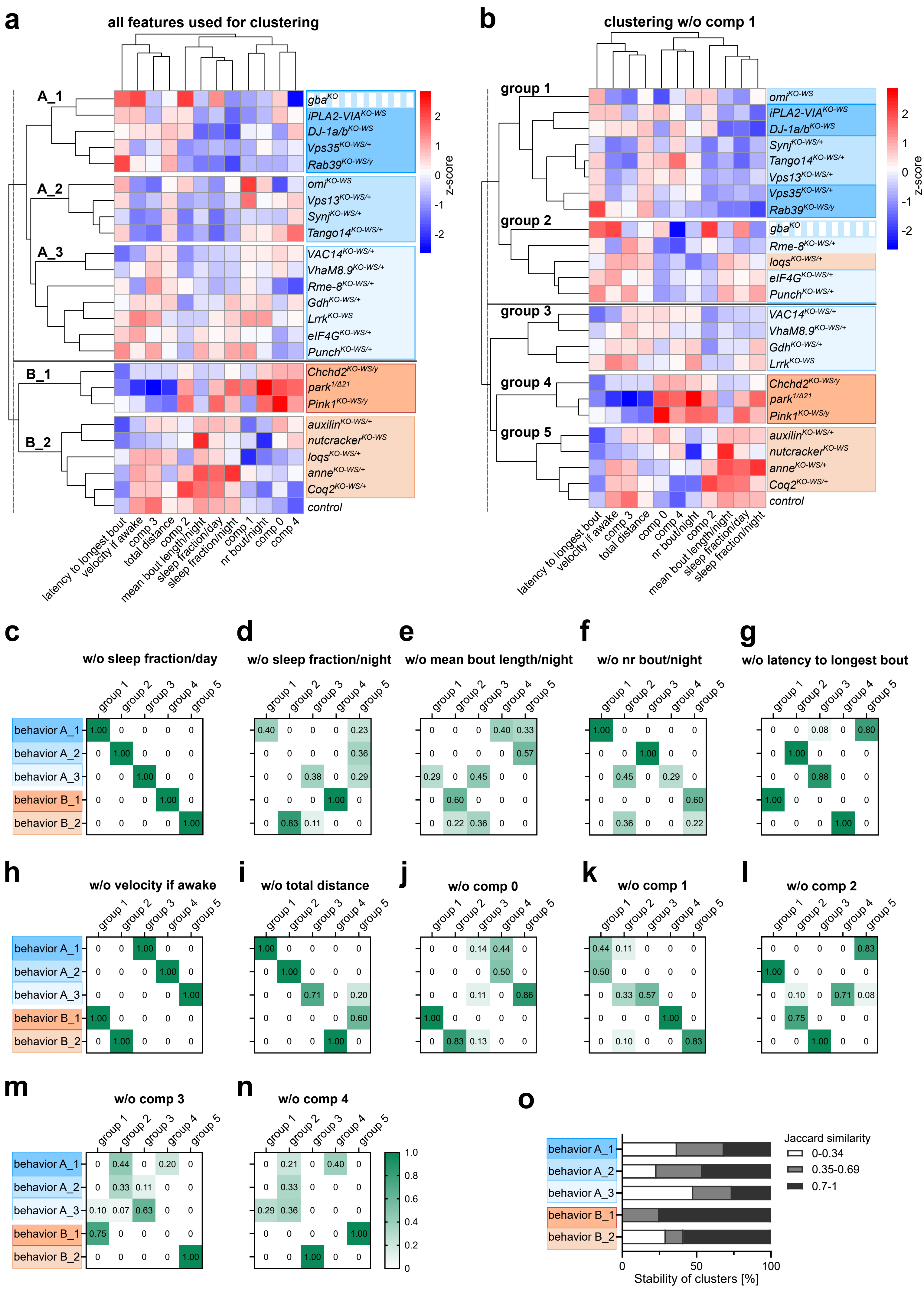

Supplementary Fig.9: Stability of behavior-defined groups in the absence of one behavioral feature at a time, *related to Fig.2*. (a) Original hierarchical clustering of Parkinsonism mutants based on their scaled behavior data reveal 2 behavior groups (A in blue, B in orange) with 3 an 2 subgroups each (dark and light shades, (behavior A1 - B2); N≥69. gba^{KO} marked in stripes as in PCA it clusters with group B (Supplementary Fig.7). (b) Example of hierarchical clustering of Parkinsonism mutants based on their scaled behavior data without "comp 1" feature resulting in group 1-5. (c-n) Heatmaps representing the Jaccard similarity index of behavior groups A1 – B2 with groups 1-5 revealed by excluding one feature each. (o) Summary of the Jaccard similarity index for each behavior group A1 – B2 highlights the stability of the originally identified clusters. Source data are provided as a Source Data file.

Supplementary Fig.10 for Fig.2

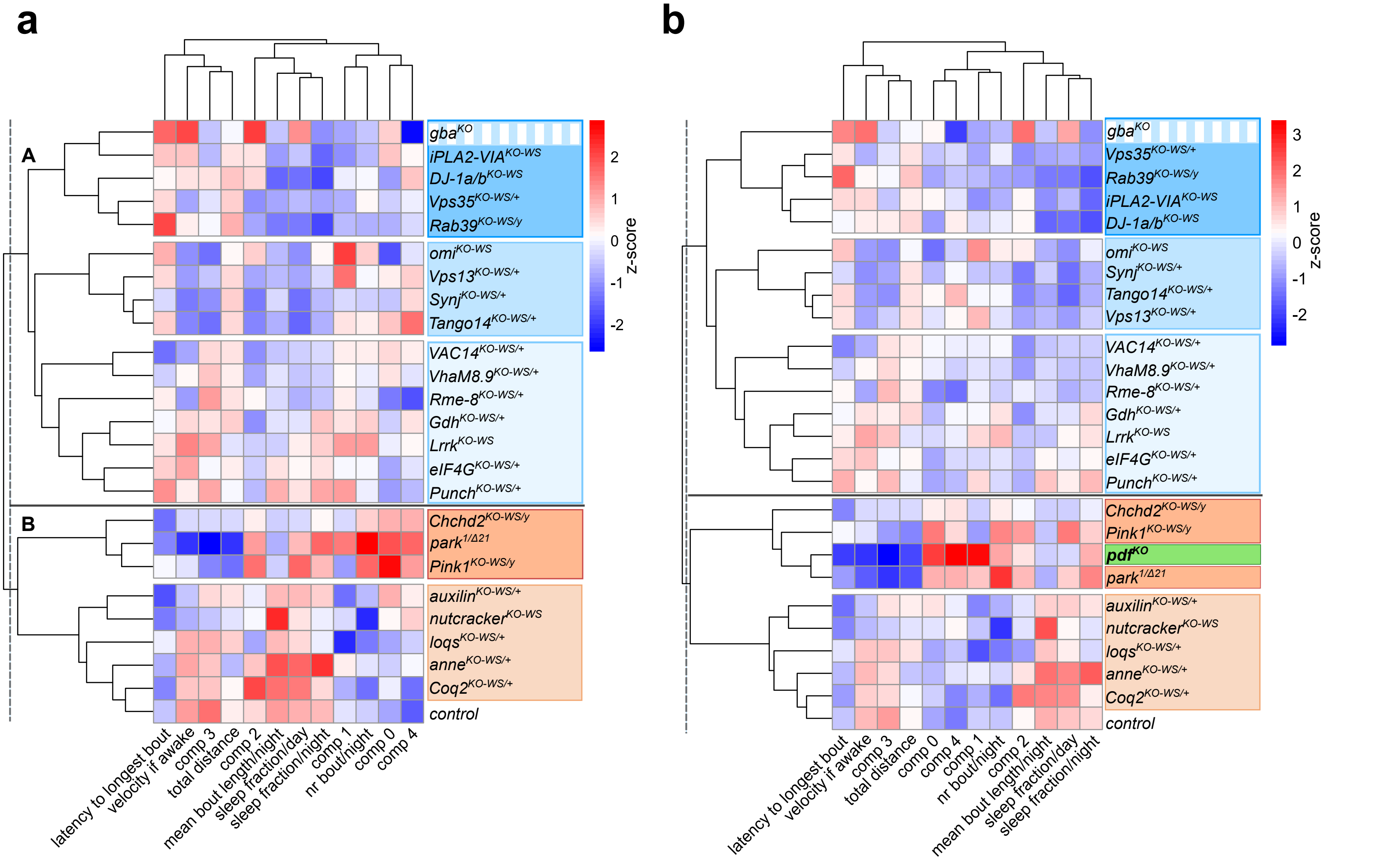

Supplementary Fig.10: The behavior features of *pdf*^{KO} flies co-cluster with behavior group B, *related to Fig.2*. (a) Original hierarchical clustering of Parkinsonism mutants based on their scaled behavior data, which reveal 2 behavior groups (A in blue, B in orange) with 3 and 2 subgroups each (dark and light shades). (b) Hierarchical clustering of Parkinsonism mutants based on their scaled behavior data including the non-Parkinsonism mutant *pdf*^{KO} flies (in green). Source data are provided as a Source Data file.

Supplementary Fig.11

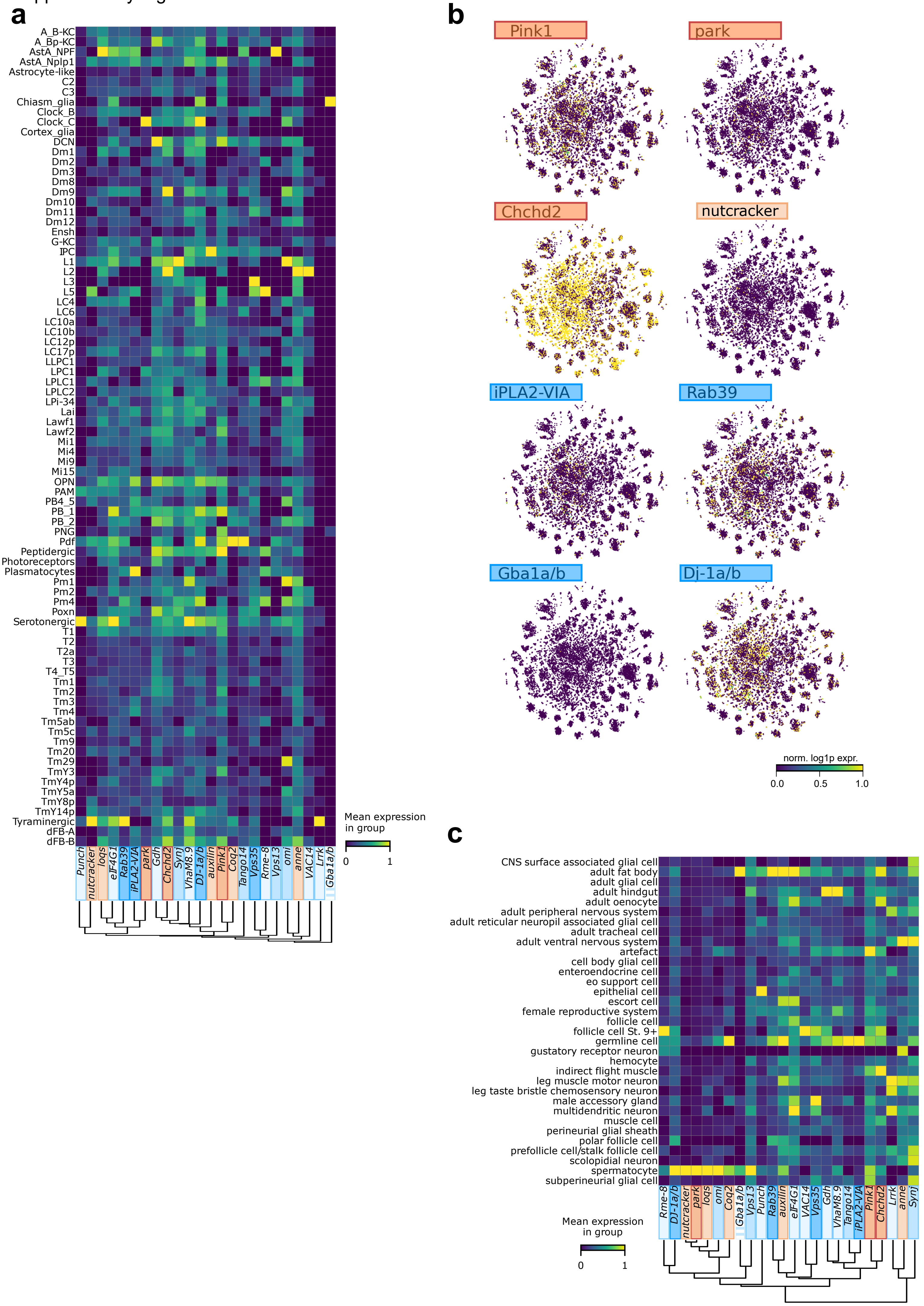

Supplementary Fig.11: Parkinsonism gene expression clustering across cell-types of the fly brain and body. (a) Heatmap and hierarchical clustering of scaled Parkinsonism gene expression across all identified cell types in single-cell RNAseq atlas[1] of brains of young (5±1 d) control flies. Parkinsonism genes are color coded according to behavior groups. (b) Example UMAPs of *Pink1*, *park*, *Chchd2*, *nutcracker*, *iPLA2-VIA*, *Rab39*, *gba1a/b* and *DJ-1a/b* normalized and logarithmized expression values. (c) Heatmap and hierarchical clustering of scaled Parkinsonism gene expression across all identified cell types of the fly body in single-cell RNAseq atlas[2]. Parkinsonism genes are color coded according to behavior groups.

Supplementary Fig.12 for Fig.3

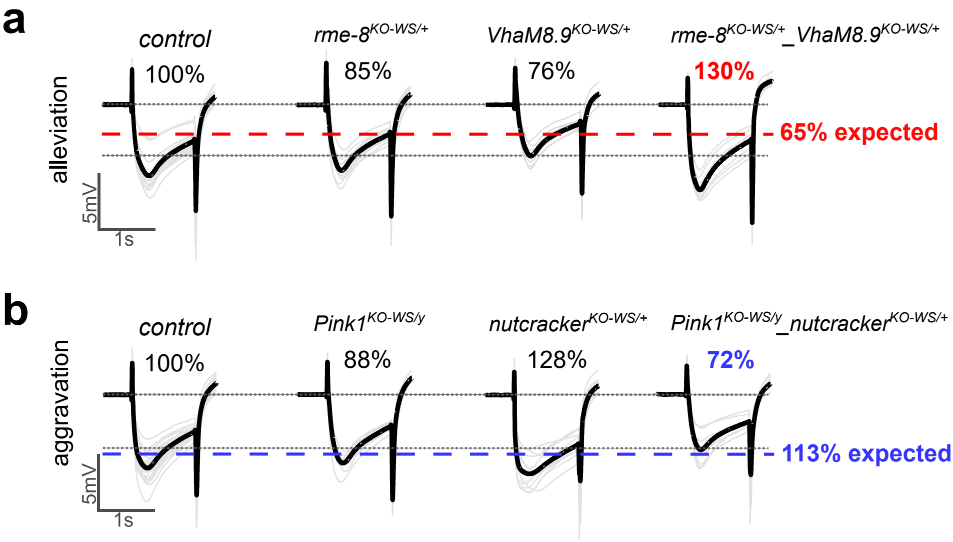

Supplementary Fig.12: Representative ERG traces of an alleviating and aggravating genetic interactions, *related to Fig.3*. (a) The ERG amplitude of double heterozygous mutants of *Rme-8* and *Vham8.9* is rescued, while (b) the ERG amplitude of the double heterozygous mutants of *Pink1* and *nutcracker* show an aggravated ERG amplitude compared to the single heterozygous mutants. Dashed lines indicate base line and 100%, respectively, while the bold lines represent the expected ERG amplitudes.

Supplementary Fig.13 for Fig 4

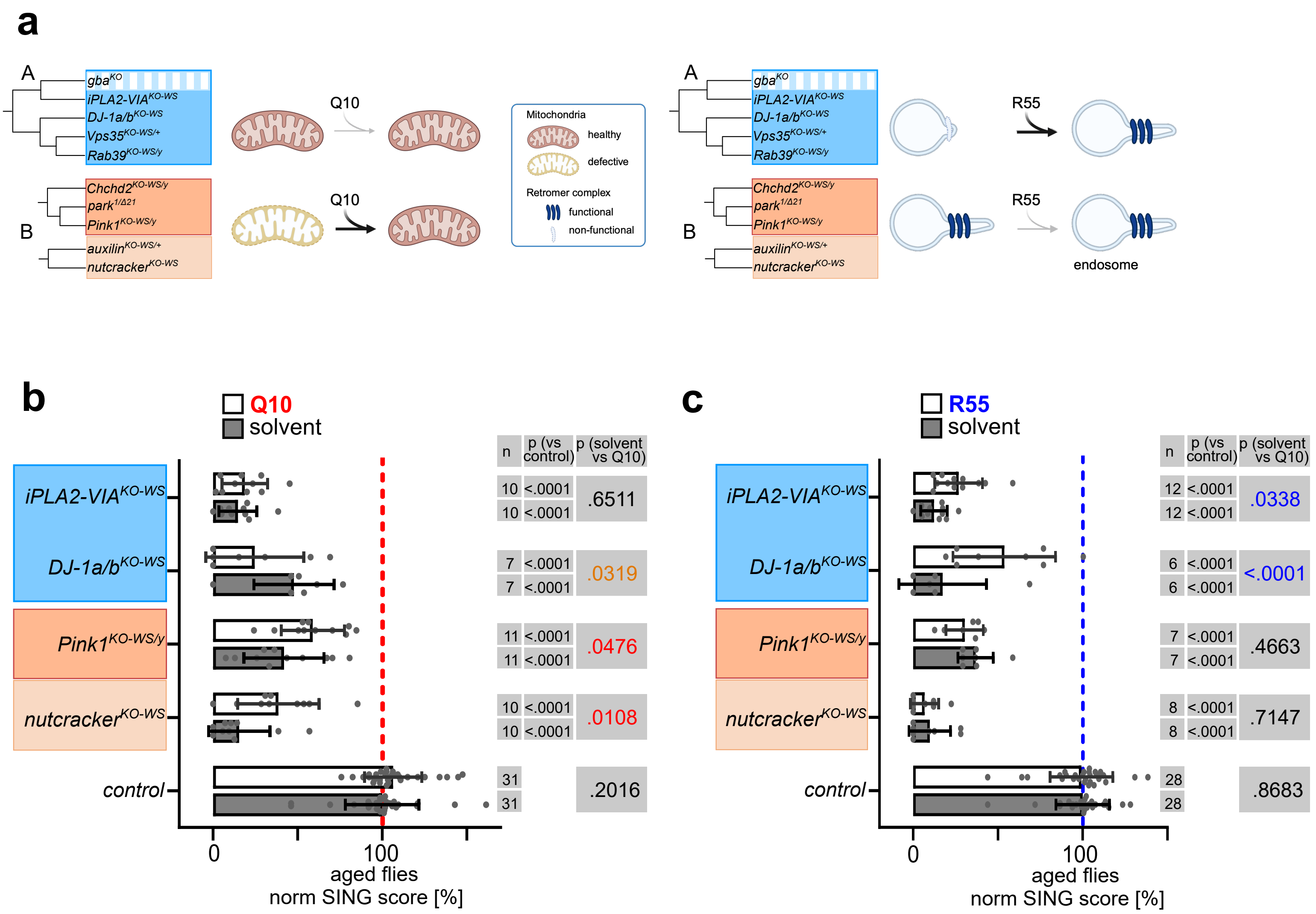

Supplementary Fig.13: Subgroup specific treatments alleviate dopaminergic neuron defects, *related to Fig.4*. (a) Schematic of experimental approach: Q10 treatment is expected to alleviate mitochondrial defects of mutants in behavior group B, but not in group A. Endolysosomal defects of mutants in group A are expected to be rescued by stabilizing the retromer complex with R55, chemical chaperone, leaving mutants in group B unaffected. Figure was created in BioRender. Verstreken, P. (2026) https://BioRender.com/y0q8cme[3] (b) SING quantification of aged flies treated with Q10 or its solvent normalized to controls with solvent treatment. Points represent groups of animals and N≥7. (c) SING quantification of aged flies treated with R55 or its solvent relative to controls with solvent treatment. (b,c) Points represent groups of animals. Bars: mean ± SD; Two-way ANOVA with Tukey's multiple comparison, n and p values are indicated in the graph; red and blue p-values indicate a significant increase, while orange represents a significant reduction. Source data are provided as a Source Data file.

Supplementary Table 1: Overview of genes with mutations in familial Parkinsonism conserved in *D. melanogaster*, *related to Fig.1.*

hGene name	dGene name	inheritance	OMIM	disease onset	Classification and additional features
ATP13A2 (PARK9)	*anne*	AR	606693	juvenile	juvenile-onset atypical Parkinson's disease, Kufor-Rakeb syndrome; dystonia NBIA, dementia, spasticity
ATP6AP2	*VhaM8.9*	XLR	300911	variable	X-linked Parkinsonism; Spasticity, seizures
CHCHD2 (PARK22)	*Chchd2*	AD	616710	adult	autosomal dominant Parkinson's disease
COQ2‡	*Coq2*	AD, AR	146500	adult	Parkinsonism; MSA susceptibility
DJ-1 (PARK7)	*DJ-1a, DJ-1b*	AR	606324	early	early-onset Parkinson's disease; seizures
DNAJC13 (PARK21)	*Rme-8*	AD	616361	adult	adult-onset Parkinsonism
DNAJC6 (PARK19A/B)	*auxilin*	AR	615528	A: juvenile<br>B: early	A: juvenile Parkinson's disease; mental retardation and seizures<br>B: early Parkinson's disease; typical PD features
eIF4G1 (PARK18)‡	*eIF4G*	AD	614251	adult	adult-onset Parkinson's disease
HTRA2 (PARK13)‡	*omi*	AD	610297	adult	autosomal dominant Parkinson's disease
LRRK2 (PARK8)	*Lrrk*	AD	607060	adult	autosomal dominant Parkinson's disease
parkin (PARK2)	*park*	AR	600116	juvenile	juvenile Parkinson's disease
PINK1 (PARK6)	*Pink1*	AR	605909	early	early-onset Parkinson's disease
PLA2G6 (PARK14)	*iPLA2-VIA*	AR	612953	adult	adult-onset dystonia-Parkinsonism with NBIA; extrapyramidal features, psychiatric/cognitive issues, seizures
Rab39B	*Rab39*	XLR	311510	early	X-linked Parkinsonism; Waisman syndrome: mental retardation, seizures
Synj1 (PARK20)	*Synj*	AR	615530	early	early-onset Parkinson's disease; seizures, cognitive decline, dystonia
VPS35 (PARK17)	*Vps35*	AD	614203	adult	Late-onset Parkinson's disease
PRKRA	*loqs*	AR	612067	early	dystonia-Parkinsonism
FBXO7 (PARK15)	*nutcracker*	AR	260300	early	parkinsonian-pyramidal syndrome, spasticity, hyperreflexia
VAC14	*CG5608 (VAC14*)*	AR	617054	juvenile	Childhood-onset striatonigral degeneration; dystonia
VPS13C (PARK23)	*Vps13*	AR	616840	early	early-onset Parkinson's disease; cognitive decline, visual hallucinations
Gch1	*Punch*	AD, AR	128230	juvenile	DOPA-responsive dystonia
GLUD2	*Gdh*	Risk gene	300144	early	modifier for Parkinson's disease
GBA	*gba 1a & 1b (gba*)*	Risk gene	168600	adult	Gaucher disease; susceptibility to PD with cognitive decline, visual hallucinations and psychiatric symptoms, seizures
NUS1‡	*Tango14*	Risk gene	[4]	early	candidate gene for Parkinson's disease

‡further replication in patients still awaiting; *Nomenclator in paper, NBIA, neurodegeneration with brain iron accumulation. *SNCA* was excluded from the collection due to poor conservation in the fly genome.

Supplementary Table 2: Overview of Parkinsonism fly line characteristics, *related to Fig.1.*

Fly lines	Viability		Fertility		Max age tested
	het	hom	het	hom	(days after eclosion)
anne^{KO-WS}	**A**	<L3	+	n/a	45
VhaM8.9^{KO-WS}	**A**	<L3	+	n/a	45
Chchd2^{KO-WS}	A	**A**	+	+	45
Coq2^{KO-WS}	**A**	<L3	+	n/a	45
DJ-1a/b^{KO-WS}	A	**A**	+	+	45
Rme-8^{KO-WS}	**A**	<L3	+	n/a	45
auxilin^{KO-WS}	**A**	<L3	+	n/a	45
eIF4G^{KO-WS}	**A**	<L3	+	n/a	45
omi^{KO-WS}	A	**A**	+	-	45
Lrrk^{KO-WS}	A	**A**	+	+	45
park$^{1/\Delta 21}$	A	**A** (transhet)	+	- [5]	45
Pink1^{KO-WS}	A	**A** (hemi)	+	- (hemi)	25
iPLA2-VIA^{KO-WS}	A	**A**	+	+	27
Rab39^{KO-WS}	A	**A**	+	+	45
Synj^{KO-WS}	**A**	<L3	+	n/a	45
Vps35^{KO-WS}	**A**	<L3	+	n/a	45
loqs^{KO-WS}	**A**	<L3	+	n/a	45
nutcracker^{KO-WS}	A	**A**	+	-	15
VAC14^{KO-WS}	**A**	<L3	+	n/a	45
Vps13^{KO-WS}	**A**	<L3	+	n/a	45
Punch^{KO-WS}	**A**	<L3	+	n/a	45
Gdh^{KO-WS}	**A**	<L3	+	n/a	45
gba^{KO}	A	**A**	+	+	27
Tango14^{KO-WS}	**A**	<L3	+	n/a	45

A, adulthood; hemi, hemizygous; het, heterozygous; hom, homozygous; L3, 3. larval stage; n/a, not applicable; transhet, transheterozygous; +, fertile; -, sterile; bold, genotype used for analysis.

Supplementary Table 3: Overview of fly stocks and genotypes used and abbreviated in this study, *related to Methods*.

Genotype	Stock name	abbreviation	RRID
w[1118]	Dmel\Canton-S-iso-w1118	CS^{w1118}	
w[1118] M{w+}	Dmel\Canton-S-iso-M{w+} w1118	$CS^{w1118}w^{+}$	
w[1118] FM7a	Dmel\Canton-S-iso-FM7a-B^{1}	*CS-FM7a*	
w[1118]; TM3/TM6C Tb[1] Sb[1]	Dmel\Canton-S-iso-TM3/TM6	*CS-TM3_TM6*	
w[1118]; Gla[1]/CyO	Dmel\Canton-S-iso-Gla^{1}/CyO	*CS-Gla_CyO*	
w[1118]; Gla[1]/CyO Tb[1]	Dmel\Canton-S-iso-Gla^{1}/CyO-Tb^{1}	*CS-Gla_CyO-Tb*	
w[1118]; ci[D]	Dmel\Canton-S-iso-ci^{D}	$CS\text{-}ci^{D}$	
w[1118]; TI{w[+]=white-STAR}anne[KO-WS]/ci[D]	Dmel\Canton-S-iso-TI{white-STAR}$anne^{KO\text{-}WS}$	$anne^{KO\text{-}WS}$	
w[1118]; TI{w[+]=white-STAR}VhaM8.9[KO-WS]/TM6C Tb[1] Sb[1]	Dmel\Canton-S-iso-TI{white-STAR}$VhaM8.9^{KO\text{-}WS}$	$VhaM8.9^{KO\text{-}WS}$	
w[1118] TI{w[+]=white-STAR}Chchd2[KO-WS]/FM7a	Dmel\Canton-S-iso-TI{white-STAR}$Chchd2^{KO\text{-}WS}$	$Chchd2^{KO\text{-}WS}$	
w[1118]; TI{w[+]=white-STAR}DJ-1a[KO-WS]/CyO Tb[1]	Dmel\Canton-S-iso-TI{white-STAR}DJ-$1a^{KO\text{-}WS}$	$DJ\text{-}1a^{KO\text{-}WS}$	
w[1118]; TI{w[+]=white-STAR}DJ-1b[KO-WS]/TM6C Tb[1] Sb[1]	Dmel\Canton-S-iso-TI{white-STAR}DJ-$1b^{KO\text{-}WS}$	$DJ\text{-}1b^{KO\text{-}WS}$	
w[1118]; TI{w[+]=white-STAR}DJ-1a[KO-WS]/CyO Tb[1]; TI{w[+]=white-STAR}DJ-1b[KO-WS]/TM6C Tb[1] Sb[1]	Dmel\Canton-S-iso-TI{white-STAR}DJ-$1a^{KO\text{-}WS}$- TI{white-STAR}DJ-$1b^{KO\text{-}WS}$	$DJ\text{-}1a/b^{KO\text{-}WS}$	
w[1118]; TI{w[+]=white-STAR}Rme-8[KO-WS]/CyO Tb[1]	Dmel\Canton-S-iso-TI{white-STAR}Rme-$8^{KO\text{-}WS}$	$Rme\text{-}8^{KO\text{-}WS}$	
w[1118]; TI{w[+]=white-STAR}auxilin[KO-WS]/TM6C Tb[1] Sb[1]	Dmel\Canton-S-iso-TI{white-STAR}$auxilin^{KO\text{-}WS}$	$auxilin^{KO\text{-}WS}$	
w[1118]; TI{w[+]=white-STAR}eIF4G1[KO-WS]/ci[D]	Dmel\Canton-S-iso-TI{white-STAR}$eIF4G^{KO\text{-}WS}$	$eIF4G^{KO\text{-}WS}$	
w[1118]; gmRPL11/CyO Tb[1]	Dmel\Canton-S-iso-gmRPL11	*gmRPL11*	FlyBase ID: FBal0230528
w[1118]; TI{w[+]=white-STAR}omi[KO-WS]/ TM6C Tb[1] Sb[1]	Dmel\Canton-S-iso-TI{white-STAR}$omi^{KO\text{-}WS}$	-	
w[1118]; gmRPL11; TI{w[+]=white-STAR}omi[KO-WS]/ TM6C Tb[1] Sb[1]	Dmel\Canton-S-iso-gmRPL11-TI{white-STAR}$omi^{KO\text{-}WS}$	$omi^{KO\text{-}WS}$	
w[1118]; TI{w[+]=white-STAR}Lrrk[KO-WS]/TM6C Tb[1] Sb[1]	Dmel\Canton-S-iso-TI{white-STAR}$Lrrk^{KO\text{-}WS}$	$Lrrk^{KO\text{-}WS}$	
w[1118] TI{w[+]=white-STAR}Pink1[KO-WS]/FM7a	Dmel\Canton-S-iso-TI{white-STAR}$Pink1^{KO\text{-}WS}$	$Pink1^{KO\text{-}WS}$	
w[1118]; TI{w[+]=white-STAR2}iPLA2-VIA[KO-WS]/TM6C Tb[1] Sb[1]	Dmel\Canton-S-iso-TI{white-STAR2}iPLA2-$VIA^{KO\text{-}WS}$	$iPLA2\text{-}VIA^{KO\text{-}WS}$	

w[1118] TI{w[+]=white-STAR}Rab39[KO-WS]/FM7a	Dmel\Canton-S-iso-TI{white-STAR}Rab39^{KO-WS}	*Rab39*$^{KO-WS}$	
w[1118]; TI{w[+]=white-STAR}Synj[KO-WS]/CyO Tb[1]	Dmel\Canton-S-iso-TI{white-STAR}Synj^{KO-WS}	*Synj*$^{KO-WS}$	
w[1118]; TI{w[+]=white-STAR}Vps35[KO-WS]/CyO Tb[1]	Dmel\Canton-S-iso-TI{white-STAR}Vps35^{KO-WS}	*Vps35*$^{KO-WS}$	
w[1118]; TI{w[+]=white-STAR}loqs[KO-WS]/CyO Tb[1]	Dmel\Canton-S-iso-TI{white-STAR}loqs^{KO-WS}	*loqs*$^{KO-WS}$	
w[1118]; TI{w[+]=white-STAR2}nutcracker[KO-WS]/TM6C Tb[1] Sb[1]	Dmel\Canton-S-iso-TI{white-STAR2}nutcracker^{KO-WS}	*nutcracker*$^{KO-WS}$	
w[1118]; TI{w[+]=white-STAR2}CG5608[KO-WS]/TM6C Tb[1] Sb[1]	Dmel\Canton-S-iso-TI{white-STAR2}CG5608^{KO-WS}	*VAC14*$^{KO-WS}$	
w[1118]; TI{w[+]=white-STAR}Vps13[KO-WS]/CyO	Dmel\Canton-S-iso-TI{white-STAR}Vps13^{KO-WS}	*Vps13*$^{KO-WS}$	
w[1118]; TI{w[+]=white-STAR}Punch[KO-WS]/CyO Tb[1]	Dmel\Canton-S-iso-TI{white-STAR}Punch^{KO-WS}	*Punch*$^{KO-WS}$	
w[1118]; TI{w[+]=white-STAR2}Gdh[KO-WS]/TM6C Tb[1] Sb[1]	Dmel\Canton-S-iso-TI{white-STAR2}Gdh^{KO-WS}	*Gdh*$^{KO-WS}$	
w[1118]; TI{w[+]=white-STAR2}gba1a CG31413 gba1b[KO-WS]/TM6C Tb[1] Sb[1]	Dmel\Canton-S-iso-TI{white-STAR2}gba1a,CG31413,gba1b^{KO-WS}	*Gba1a-CG31416-Gba1b*$^{KO-WS}$	
w[1118] M{w[+]}; TI{ReC-CG31413}gba1a CG31413 gba1b[KO]/TM6C Tb[1] Sb[1]	Dmel\Canton-S-iso-M{w[+]}-TI{ReC-CG31413}gba1a,gba1b^{KO}	*gba*$^{KO\text{-}w+}$	
w[1118]; TI{ReC-CG31413}gba1a CG31413 gba1b[KO]/TM6C Tb[1] Sb[1]	Dmel\Canton-S-iso-TI{ReC-CG31413}gba1a,gba1b^{KO}	*gba*^{KO}	
w[1118]; TI{w[+]=white-STAR2}Tango14[KO-WS]/CyO	Dmel\Canton-S-iso-TI{white-STAR2}Tango14^{KO-WS}	*Tango14*$^{KO-WS}$	
w[*]; Df(3R)Pdf[attP], TI{RFP[3xP3.cUa]=TI}Pdf[attP]		*pdf*^{KO}	BDSC_84561
w[*]; P{w[+mC]=EP}park[1]/TM3, Sb[1] Ser[1]		*park*^{1}	BDSC_34747
w[*]; park[Delta21]/TM3, P{w[+mC]=GAL4-Kr.C}DC2, P{w[+mC]=UAS-GFP.S65T}DC10, Sb[1]		*park*$^{\Delta 21}$	BDSC_ 51652

Supplementary Table 4: List of primer pairs used for quantitative RT PCR, *related to Methods*

F1_qPCR_anne	CGTCGAGCAACCTTAAGACA
R1_qPCR_anne	ACTGACAGTTGAAGGACGTTT
F2_qPCR_vham8,9	GTTTATAGCGGCCATCAATGC
R2_qPCR_vham8,9	CGTTGCCCTTGAAACTGATTG
F2_qPCR_CHCHD2	CTCAGCCATTGGACACAC
R2_qPCR_CHCHD2	CTGGATGAACTGTTTGAG
F2_qPCR_Coq2	GCAGCAGATCTATTCCCTCAA
R2_qPCR_Coq2	GAATGAGTCCTACTTGGTGGTT
F2_qPCR_dja	CCATATCCGCCGATGTTCTAC
R2_qPCR_dja	GGCACGATGACCACAGAT
F2_qPCR_djb	AGCGGAGGAGATGGAGTT
R2_qPCR_djb	CATCCCGCGAGCACTTC
F2_qPCR_rme-8	GACAAGCTTGACCTGACTAACA
R2_qPCR_rme-8	ATGGTAATCACGAAGTCATGGG
F1_qPCR_aux	GTTGAAGTCGCTGGACACA
R1_qPCR_aux	GCACATCTTGGGCTACATACA
F1_qPCR_eIF4G1	GCAACAGGCTATACCAACTTTAC
R1_qPCR_eIF4G1	TCCGCCACTTTGTGGATTAG
F2_qPCR_omi	ATCGAGGACGTGGATCAGA
R2_qPCR_omi	TAGCCGCATCACCGAAAG
F2_qPCR_lrrk	CAGGAGCAAACACCCTTCTAT
R2_qPCR_lrrk	TCCAGCATCCAGCAATCG
F1_qPCR_iPLA2	TCGTCAGCATTGTGAAAGAGTA
R1_qPCR_iPLA2	GACAGCGTTTAGGAGGGATTAT
F1_qPCR_Rab39	GCTCGCTGCTCAAATTCTTC
R1_qPCR_Rab39	TGTGTGCCGTCCTTCATT
F1_qPCR_Synj	CGATCTGCAGCAAGAACAATTC
R1_qPCR_Synj	CCACTAGCAGGACATCAATGG
F2_qPCR_VPS35	AAGTGCGCCAGCACCAT
R2_qPCR_VPS35	GGCCATGTACAGCTCGTAGTAA
F1_qPCR_loqs	GCAATGCCAATGCCACAG
R1_qPCR_loqs	CATGCACATTTCCTGCAACC
F1_qPCR_Gdh	CATCAACGAGGCCAACTACA
R1_qPCR_Gdh	GAAGCCCTGGACGATGAAG
F2_qPCR_nutcracker	AACAAGTGGTCGGCACAA
R2_qPCR_nutcracker	CTGGGTGCTCTTCTCTTGTATC
F4_qPCR_punch	AAGTGCACGTTCCACCA
R4_qPCR_punch	CGCCCAAGAGTAGACGATAC
F1_qPCR_Tango14	TCCCAGCAAACTGGAGAAC
R1_qPCR_Tango14	GTCGGAGGCGCTGATTT
F2_qPCR_gba1a	AAGGACATCACCCTGTTTGG
R2_qPCR_gba1a	TCGAGTGCGGTTCATCATTT
F2_qPCR_gba1b	CCCGAGATGCCAGATATGAA

R2_qPCR_gba1b	CAAATCGCAGGGCAACTG
F2_qPCR_Vac14	ACATCACGGGACGCAAA
R2_qPCR_Vac14	CCATGCCCTGGACATACTT
F2_qPCR_VPS13	TGTGCTGAACAAGGTGCT
R2_qPCR_VPS13	TCTCCCGTATCTTGAGGTTCT
F6_qPCR_parkin	CCAGCAATGTCACCATCAAAG
R6_qPCR_parkin	GCGTGTGCAGACCATGT

Supplementary Document 1: Cloning and primer details for Parkinsonism KO *Drosophila* models, *related to Methods*

Cloning of *pWhite-STAR2*

Primers to generate SV40 poly(A)-tail

F_hifi_SV40: GGTGGTCCCGTCGAAAGCCGAGGATCCAGACATGATAAG

R_hifi_SV40: ATGGACGAGCTGTACAAGTAAGGATCTTTGTGAAGGAAC

gBlock_P3XGFP:
CGGCCGCTATAACTAGTAACTCGAGGGATCTAATTCAATTAGAGACTAATTCAATTAGAG
CTAATTCAATTAGGATCACAGCTTATCGATTTCGAACCCTCGACCGCCGGAGTATAAATA
GAGGCGCTTCGTCTACGGAGCGACAATTCAATTCAAACAAGCAAAGTGAACACGTCGCT
AAGCGAAAGCTAAGCAAATAAACAAGCGCAGCTGAACAAGCTAAACAATCGGCAAAAT
GGTGAGCAAGGGCGAGGAGCTGTTCACCGGGGTGGTGCCCATCCTGGTCGAGCTGGACG
GCGACGTAAACGGCCACAAGTTCAGCGTGTCCGGCGAGGGCGAGGGCGATGCCACCTAC
GGCAAGCTGACCCTGAAGTTCATCTGCACCACCGGCAAGCTGCCCGTGCCCTGGCCCACC
CTCGTGACCACCCTGACCTACGGCGTGCAGTGCTTCAGCCGCTACCCCGACCACATGAAG
CAGCACGACTTCTTCAAGTCCGCCATGCCCGAAGGCTACGTCCAGGAGCGCACCATCTTC
TTCAAGGACGACGGCAACTACAAGACCCGCGCCGAGGTGAAGTTCGAGGGCGACACCCT
GGTGAACCGCATCGAGCTGAAGGGCATCGACTTCAAGGAGGACGGCAACATCCTGGGGC
ACAAGCTGGAGTACAACTACAACAGCCACAACGTCTATATCATGGCCGACAAGCAGAAG
AACGGCATCAAGGTGAACTTCAAGATCCGCCACAACATCGAGGACGGCAGCGTGCAGCT
CGCCGACCACTACCAGCAGAACACCCCCATCGGCGACGGCCCCGTGCTGCTGCCCGACAA
CCACTACCTGAGCACCCAGTCCGCCCTGAGCAAAGACCCCAACGAGAAGCGCGATCACA
TGGTCCTGCTGGAGTTCGTGACCGCCGCCGGGATCACTCTCGGCATGGACGAGCTGTACA
AGTAA

Cloning of pReC-CG31413

gBlocks to generate pReC

gBlock_attB-MCS-SV40:
GTTGTAAAACGACGGCCAGTGAATTCCCGCGGTGCGGGTGCCAGGGCGTGCCCTTGGGCT
CCCCGGGCGCGTACTCCACTCGAGCGCTAGCTAATCGTCTAGatgcGAAGAGCGAGCTCGG
TAGATCCGATATCCTGCAGGCATGCAAGCTTGGCGTAATCATGGCTCTTCgtaaGGATCTTT
GTGAAGGAACCTTACTTCTGTGGTGTGACATAATTGGACAAACTACCTACAGAGATTTAA
AGCTCTAAGGTAAATATAAAATTTTTAAGTGTATAATGTGTTAAACTACTGATTCTAATTG
TTTGTGTATTTTAGATTCCAACCTATGGAACTGATGAATGGGAGCAGTGGTGGAATGCCT
TTAATGAGGAAAACCTGTTTTGCTCAGAAGAAATGCCATCTAGTGATGATGAGGCTACTG
CTGACTCTCAACATTCTACTCCTCCAAAAAAGAAGAGAAAGGTAGAAGACCCCAAGGAC
TTTCCTTCAGAATTGCTAAGTTTTTTGAGTCATGCTGTGTTTAGTAATAGAACTCTTGCTTG
C

gBlock_SV40-attB:
AGTAATAGAACTCTTGCTTGCTTTGCTATTTACACCACAAAGGAAAAAGCTGCACTGCTA
TACAAGAAAATTATGGAAAAATATTTGATGTATAGTGCCTTGACTAGAGATCATAATCAG
CCATACCACATTTGTAGAGGTTTTACTTGCTTTAAAAAACCTCCCACACCTCCCCCTGAAC
CTGAAACATAAAATGAATGCAATTGTTGTTGTTAACTTGTTTATTGCAGCTTATAATGGTT
ACAAATAAAGCAATAGCATCACAAATTTCACAAATAAAGCATTTTTTTCACTGCATTCTA

GTTGTGGTTTGTCCAAACTCATCAATGTATCTTATCATGTCTGGATCCGTGGAGTACGCGC
CCGGGGAGCCCAAGGGCACGCCCTGGCACCCGCACCGCGGGGTACCGTCATAGCTGTTTC
CTGTGTGAAATTGTTATCCGCTCACAATTCCACACAACATACGAGCCGGAAGCATAAAGT
GTAAAGCCTGGGGTGCCTAATGAGTGAGCTAACTCACATTAATTGCGTTGCGCTCACTGC
CCGCTTTCCAGTCGGGAAACCTGTCGTGCCAGCTGCATTAATGAATCGGCCAACGCGCGG
GGAGAGGCGGTTTGCGTATTGGGCGCACATCCGCTTCCTCGCTCACTGACTCGCTG

Primers to amplify the CG31413 genomic region

F_hifi_CG31413: CTCCCCGGGCGCGTACTCCACAGAAGATGGTTGGTTTCGG

R_hifi_CG31413: TACCGAGCTCGCTCTTCgcatCTCGGGCCAAAAACAATGC

gRNA's used to target genes of interest and make the pCFD4 constructs

Gene	*Construct*	*5'-3' gRNA 1*	*5'-3' gRNA 2*
ATP13A2	pCFD4-Td_gRNA:ATP13A2	ACAATATCAAGTTTGCGCCT	CTACTTTTGAATTGGGCCG
Vham8.9	pCFD4-Td_gRNA: Vham8.9	GTTATCGAATGGGTATCAGT	GCATTTTACTTGATTATAGA
CHCHD2	pCFD4-Td_gRNA:CHCHD2	GTTTTGCCAGTCACAACGGG	AACATGAATGTCTCGAACGC
Coq2	pCFD4-Td_gRNA:Coq2	ATGCGTCGTGGGTTTCTCTG	TTCTGTAAGGTAATATAACA
Dj1-a	pCFD4-Td_gRNA: Dj1-a	CGTTTTTGTTTTACAAGGGA	TTGTGCCGCTCCCACGGCGC
Dj1-b	pCFD4-Td_gRNA: Dj1-b	GCTTTGAGTTTTCCTTATCG	GCCGGACACCTCGCTGGCCC
rme-8	pCFD4-Td_gRNA: rme-8	AGACCAGTCATGAATTGCCG	TTTAGTTCCGAGCTCTCCGT
auxilin	pCFD4-Td_gRNA: Auxilin	GCTCTTATCGATGTGGCGCG	TCACGGTTACGCGTTACCGC
eIF4G1	pCFD4-Td_gRNA: eIF4G1	TTGATATACAGCTACCAGAG	GCAGCATAGCCATCCCACTG
ntc	pCFD4-Td_gRNA: ntc	ATATATTTGATAAGACACT	AAACTATCCTCAAAGATAA
Gba1a_1b	pCFD4-Td_gRNA: Gba1a_1b	AAACCAACCATCTTCTGGT	ATTTGAGGAGTCTCATGGCC
punch	pCFD4-Td_gRNA:punch	TAGTTAGTCAATGAACTAGG	CCGGCGTCTTGATCAGTCCC
Gdh	pCFD4-Td_gRNA:Gdh	AGTCGACTGATCAAGCCGGT	ATGAAAGTGCATCATCCTAA
Omi	pCFD4-Td_gRNA:Omi	GTATTGCTGTCGTTTCTAGT	TCCCGCCACCATCGAGGACG
Lrrk	pCFD4-Td_gRNA:Lrrk	CAGTGCGACATAAAACGGCC	GCTCTCGTAACCAGACTGGC
Tango14	pCFD4-Td_gRNA:Tango14	TAGCAACGTAATCGATTCC	CGTAGGCACATCGTTCGCG
Pink1	pCFD4-Td_gRNA: Pink1	TGCGGGGCGAAGTGTGGGGG	ATTAAACGGTGATCCCGACG
iPLA2-VIA	pCFD4-Td_gRNA: iPLA2-VIA	TTGCAAGCCCCTACCATTGG	CCTTGGCTGATAATCCCTCC
Loqs	pCFD4-Td_gRNA: Loqs	AACAAACTTACGATCATGCC	TCCGCTGGTCCGTCGGTGAC

Rab39	pCFD4-Td_gRNA: Rab39	CTACTATCGATTACTTTTGC	CTGCTCAAATTCTTCACAGA
Synj	pCFD4-Td_gRNA:Synj	GTACTTCGTTTTTAAGGTGT	AGCTCTCTCAACACAGTGGT
VPS35	pCFD4-Td_gRNA:VPS35	GTATGGTGGTGACTTCTCTG	GAAGTTGTATAAAGACGCTT
VPS13	pCFD4-Td_gRNA:VPS13	TCTCTCCGTTCCATCTGTT	GAATGGTGTCATCCTCTCC
VAC14	pCFD4-Td_gRNA:VAC14	ATTAGGATGACGAATCTCGG	TATTTGTGACCATTAGTCAT

Cloning of the donor constructs:

The homology arm gBlocks undergo modifications to ensure compatibility with the manufacturing prerequisites. Within these modified gBlocks, specific mutations are introduced, highlighted in green, strategically designed to prevent Cas9-mediated cleavage within the donor plasmid.

Gene: ATP13A2, Constuct: pWhite-STAR_ATP13A2

gBlock_ATP13A2_LHA:
AACAGGGTAATGGTACCTACGTTCCGCAAGAATATCTGCGGTTTCAGGTATTTTTAATAG
CTTCAAAAACCAACAAGCGTCCGATGGTGCCGTATTGTTCGCGTCTAGTCACATTAATAA
TACCCGTTACTCGTAGATTAAAAGGCTACACTAGATTTGTTGAAAAGTTTGTAACATGTA
GAAGGAAGCGTTTCCAACCATATAAAGTATATATTCTATATACATACATACATATGTATA
TTCATGTTTTTATTAATTTTCGCGTCGACTTTAGTCTGCCAGCTGAATGCTTGGCGAAAAG
TTTTAAGTAGGTTTTTAAGGCAGTTAAAGGATGAATAATGATTTAAGACATGGCGTTTAA
ATTTGATTTGTTGGCTTCGAGTAAATTGCTTTTCTATATATCGAAAATTCTCAGATATGAT
TACTGACTGCATTTTAAATTACCTACTAATTAAAAGTTTAAATAGGTGACTAATGTATGTT
CATATTTTTAAATAAATATGTATATGCATTTGTATGTATATGAAGCAACAAATCTGTTTTC
AGCGTTGTTGTATTAATATATGCATACATACATGTATTGATCAGCACAAGCCAGCTGCAC
AAAAATTTGAATGAATATACATATGTACATATGTACGGTACATGTGTATGCATTAATTGA
TTGTTTCTCATTGTATTTGTTATGACCATCTCGAATGTGTGTATAAGTGGCTCAAAATTCA
TGTATTATGTTTTCACTTTGTATGTACATATGTATGTATGGAAAGCTTCATATATGAGTGA
ATTATTAGGTGTAACGCACAATATTTCTATTAAAGATTTGTTCTGTTGGCAACAGTGTCCC
AATTACGTACAATTGAAAAGCCCCATAATCATATAAGCGAATAAAAAACGGGTACGAAA
AGAAAAGTGTGAGTGCTCTAGCGAACGAACAATATCAAGTTCGCGCTTTGGATTCTTATC
ATTTGTCTTTTATCTATTGATGTTGCCTTTTCGATTAGGCGTTTAAATAGCTGTTCGTGGCA
ACAACTTTTTCTTTAAATGTGCCTTGATTTTTTTGGTAAAAGGTCACAGTTAATTGCCTTA
ACAATGTTAGTTTCAAGACATTAACGGATTAAATTTTAGCTATGCAATTTAACAATATTTT
TTGTTTCAACTATAGGAGTGTAATTGATTTAACTTTACTGTTCTTATAATTATACAAAATA
AAACAGACAAAAATAAGCCAAGGTAGGCGTACTTAAATACATACATAGATAGAAGAAAA
TAGCAAATTATGACACCCCACAATGATTCAAAAGGTATGCACTCGATATGCGGCCGCGTA
GTGC
gBlock_ATP13A2_RHA:
CGGCCGCTATAACTAGTAACCATTCATCAGGACACTTTTGGAAAAGGAACAGCAATCCAT
AGAAAGAACTCATATAGAATGCGATCATGTAGAAAACGTCCTTCAACTGTCAGTTCATTT
TACAAGTGCCCAATTCAAAAGTACGTGCCATATAAACATATTAAGATACACCAATTTCCA
ACATCGTTTTTTTTTAGAATGCTCATCAATACGAATTTTTCGCTGCAAGCAATTGGTTTAT
GCTTGGAACAATAATACAAATAGATTTCAAAGGATAAATGGACTCGACCTAAATATTCCT
TGTTCATATTATCACCAACAGCGTGGATTACCTGTACATGAACAGATTTCAAGGCGAATT
GTTTTCGGAGATAATGAGATAACTGTACCATTGCGAGATTTCAAGACATTGCTGTTCTTAG
AAGTACTTAATCCTTTTTACGTTTTTCAATTATTTTCTGTAATTCTTTGGTTTACATATGAT
TACTATTACTATGCTTGCGTAATACTCTTGATGTCAGTTTTTGGTATAACAGTGTCTGTTTT
ACAAACGAAAAAGGTAAGTATATCAATTAGAAGAACTTTATAAAATATAATTCCTATATA
TTTTTAGAATCAGGATGTGCTCCAAAAAACAGTATATAACACTGGTAATGCTTGGGTTGT
TGATCATAAAGGACTGTCTAAAGAGCTTCCAACGCGAGCGATAGTACCTGGGGACATCAT
TGAAATACCCTCATCAGGGTGTACGCTGCATTGCGATGCAATCTTAATATCAGGAAACTG
CATTCTAGATGAGTCTATGCTTACTGGTGAAAGTGTGCCAGTGACCAAAACTCCTCTACC
GTCGAAACGTGACATGATTTTTGATAAAACAGAGCATGCCAGACATACACTTTTTTGTGG
CACAAAGGTTATTCAGACTCGTTATATTGGCTCCAAAAAAGTATTAGCATTTGTAATAAA
CACTGGAAACATAACGGCAAAAGGAGAACTTATACGTTCTATTCTTTATCCtCCCCCTGTG
GACTACAAGTTTGAACAAGATTCGTACAAATTTATCCAGTTTCTGGCCATAATAGCATGT
GTAGGATTTATTTATACGCGTAGGGATAACAGGGTAATG

Gene: Vham8.9, Construct: pWhite-STAR_Vham8.9
LHA_F_Primer: AACAGGGTAATGGTACCTACTATATCGCTAATGCTCTA
LHA_R_Primer: GCACTACGCGGCCGCATATCAGTTTTGTTGTCGAAGAGTGCGAA
RHA_F_Primer: CGGCCGCTATAACTAGTAACAGATCTAAAATCTATAATCAAGTAAAAT
RHA_R_Primer: CATTACCCTGTTATCCCTACGACATTCGAGGGCTTGGC

Gene: CHCHD2, Construct: pWhite-STAR_CHCHD2
gBlock_CHCHD2_LHA:
AACAGGGTAATGGTACCTACATAATTTTTAGAGCCTTAACATTCGTTTTTAAATCTTAAAA
ACGTCTTAAAACAAAAATAAACTACAAAAAAAGTTCCAAGGTTTATGAAAGACGGAAGT
TTCAGTGAAGATTACGTTTAAGTATACAAAAAGGGTCACAATAACTTTTAAATACATATT
TAAGTAGTTTTCCTGCGTTAACACGTTTTTTTTAAACTGTAGATGGGCGCTATACGCACAC
ACACACACGCATAAGCACCGTAAAAAACACACGCGCGTTGTTGTTGTTGAATTGCGAGTT
TTTAAGGGTTTATACTTTCTTGAAGGTTATCCTTTTGGATAATTGGCTCAATATACGTGGA
TGTATATTGTATTAAACATATTCTTGCGCTGCAAGCAGATAAAAAAAAATATACAAAAAAA
AAGGGGAAACAAACACGTGTGACGCACACACACAGTTGCACTCGCGCGCACACACACAA
ACAAAAAGCCGCCCGTTTTTAACCTCATTTCGGTGGAAATGGCAATATTCCGCAGTTTGC
CGCAGTAAATTCACAAAACCCTTAGTATCCACACTAGTTGCCAGCGTTTAGCCGTCTTTTG
GCCATCCTCTGGCCACAATCCACACAATTAAACGTTGAAAATCCCTTACTTTGTCACAGA
AGAAATCGAGATACGATGTCAGCGCAAAGGCAAACGCCTGAAAAAGAACATGTTACGGC
AGTGTGCAGCAACGCCAGAGAGTCCGCTTCCGCCGACACGCACACGGTTGCATTCCGGCT
GCCAGGGCTGCGCAACAAGTGAACTATCGAACGGAGATAGGCGGCCATGAGTGGGGATT
ATTCCTCGACTTAATCACTTTTTCGAACGCGTTTGCTACTTGAGTTGCACATTTATATGTAC
TTCATAGAAATATACCCTGCTTTCAGGCATACTATTAGTTTTAAGCCACTTTTTTAATGAC
AAATTTCGAACTTTCTATCGATATGCGGCATTTCGCGGTTTTGCCAGTCACAACAAATGGC
GATAGTTGAAATTCGCATGCCAAATTTTCTTTATTACGGTCACCTTATAGAAATTTTGATA
TGCTTGAGTATTAAATGTTATTATTTCACTGACTGTAAAATAAAAAGTTATTTAAATCGTA
ATGCATATATATTTATAACATATAGTTCTTTATTTGATTTTGTAAGTGGAAGATATGCGGC
CGCGTAGTGC
gBlock_CHCHD2_RHA:
CGGCCGCTATAACTAGTAACGGTTAAACGAGCGATTAGTCATAGATCTTATTAATGGTCT
TATGAAATATTTGCCGTGGAAACGTAATTTTGAAAAGTTAAGAACTGTTTAAAAGGTTCC
CTAAATTACCACCCAAAAAAAATATTATGAACTAATCCCTAAATTCGAGACATTCATGTTT
CCGTTTTTATTCAATAATTTTTACATAGAGTCATCACTGTTCATAATTTTGCAGCGTATTGA
TTTTTAGGATTAACAATTTTTTTGTAATGGGGCAATACGTATGTAACAACTACAAATTTTT
CGACTCTAGTAACACTGATGTCTCCTATTATCGAAAAACAGTGTCTATATATATATATATA
TAAAAAAAGAAACAAAGAGTTATAAAGAACCATCAAAATGCCAAATGGATGAAAGCTAT
CGTTTTCGTAGCTTTTTTGAAGATTTTTGGAATTTTCGATTCTGGTCGTTTTCAAATGGTAC
GATTTTAGGGGATTAGAGTTTTCCGAATTCAAAAAAAAGTCTCCGGGGAGGAGCATCGAT
CCACCAGGTGTGTATGTGTGTATATATAATGTTGCATATTTGTAGTGTTCACTGCTCAATT
TGGAATTCCGCTTCAAATGTGGATTTGCCTTCGATTGTCTGGGTTTTATGGGTAAAAACGG
ATGATATAAGGGGTCATCATCTATCTTCAGAGCTTTCCGGTCTTTGTGGTCTGCGGGCCAA
GACTTTCCCGGGACTTTTTCTTGTGCAGCACGCTGGGCACCTGAACCTTTGGACTGACCAC
CGGTTGCTTGGGTGGCTTCACCTTCTTGGATGATCCCGCCGCCGCGGCGGAGCTGCGCTTG
GCCAGGAAGTAACCGTCCGTCTTGTCCTTGGTGATGCACTTCTTGCCGGGATATGCTCTCC
TGCGATAGGGGGTGATCGCGTCGTACATTTTGCTTCTGACCACATACGCCTGCTTCCAGCT
TGAGGCCACCTCCTTCTCTTTGATGCGTGGCTTGTCGAGATCCTTGAAGGGATCGGCCGCC
TTGCTAATGTCCGCTTTGCTGGTCACATGAGGCAGTTTCTGGCTCGCCACTCTGTGGTAGT
CAGTGGCCGATGATGGGGACTTCGGTTCGGATCTGGGCTTGGCTTGGGCCTTGACGATGG
CCTCCGTAGGGATAACAGGGTAATG

Gene: Coq2, Construct: pWhite-STAR_Coq2
gBlock_Coq2_LHA:
AACAGGGTAATGGTACCTACTTACAGCGGTATAATTTAACCCATGAATCTTTATTCATGA
GACACCCTGTTCGAGCATATTCAATTTGTATTATTTGTATTTGCTGAGAGCGAATAAAGCG

CTAGAGAGCGAATAATTCGTTGTGGTTTCTGGCACATAAACATACTTATACGAACTTATA
CTAGGGAGTGACTAGCCAGAGAATTCAAATTCCAAGTCGGACGAGATATTTGTTTGCTCT
CCGTGCAAATCTCATTCAGCTAAACTGGATGAGTGACTTTTAAATTACAGCGTTTAGCGC
AGTGGTAAGTGATTTTGTATCAAAATTGTTGGACACAGGAGTTGTGTAACTGCGTACTCA
CCACCATGATCATAGGGCAGGACTTGGCCGTGCTGGTGCTGTCATCCGAGCCGTGATCCA
TCTTGGGCTGCTGGTGTAGTATAGATATAGATAGAAGGCGTGGAAAATCCGACTGGCGAT
CGGGGAGGCACAGTGATAAGAGCTAGACCTGCTGAGGTGCTGAGAGCAGAGCGTTGACT
GCTTGAAACCCGCTGCTCGCGAGTGGTTTTTAAAGAAAATCGCACCTAATCCCCCGACG
CGGCGATAGGAAAGTGCGCTTATCTATGGGCAATGGGAAAGAGCGTTATCACCCAGTAG
CGGCGATACCAACGATAGTCGCCCCACACTAAGAAAGGCAGCCCAGATACAGTCATGTA
AATAAACATAAATGCATGCAGATCTCAGTGTTAGAGGTCAGATCAAAAGATATATTCTGG
GTATCAGTTGCAGATCTTTATCTCGAGAGATAAAATCCATAATTGATGCGTCGTGGGTTTC
TCTGCaaTTTGCAGCTTAATTTATGGACTCATGGCAAAGGTTCGACAATCGATTCGTGTGC
AGAATTTATTGCAATTCAGATTATCAATCATGGGCTAATACAACCCCCTGAAATTGTTGCT
GTAATACATGATGTTGAAATAATTCCATTATTTCTTCGCTTCGATCACGGTTTTATGGCTC
TAGTCGAGTACATTCATACGTAGGTATATGCCCATATTCCGGCATGGGAGCGATAGCAAC
CGTCGGCCGCCGGATCGCCGGAGGTCTGCCTTAGTCCACTTGGCTTTCCTAGAGTGTTATT
TCTTACTGTGCTATTTCTAACTGGGATAGAATGCCGCCAAAATTGATGGCCGTGCGCAAA
CCTTCTGCGGCCGTGTGCATAAATTCTACGGACATTCCTGCGCCGTGTGCCAATTTCGAAA
AATGGGCGACGTGCGCAAAACATTAACGATATTGCAGAGGGGTAAATGGTTGGGTCTAC
GCAAAGATATGCGGCCGCGTAGTGC
gBlock_Coq2_RHA:
CGGCCGCTATAACTAGTAACAATTGTTATATTACCTTACAGAAAGTCACTTATTCATCCAT
TTGGCATTTCAGATCTATTCCCTCAACATTGACAACCCCAGCGACTGCGCCAAAAAGTTC
ATATCGAACCACCAAGTAGGACTCATTCTCTTCCTCGGCATTGTTCTGGGCACCCTTCTGA
AATCAGACGAAAGCAAGAAACAGCGACAATCCTCACTAACAACATCGACGGCCAGCTCG
TACGTTCCAGCGCTGCCGCAAAAGCCAGAAGTTTTAAGCTGAGATGAGCCAGACGAGCG
TAGATTGTAGTGTTAAATTATGTTTATAGCGCGCTTTTTGATTCGCCAAAACCAAAGTCTC
TAGGTATCTGATTAGATAAAATCTTGTAAATAGGCTTATGACCATTTTTAGCTCATTTGCA
ATAGGTTAAGTCTTAAAGAATATTACTAAGTGCACACCTCACTTAAACATCAATTAATTCT
TAATTGTAAGTGCCCGCCTTGACGTAAGAACTTACGTAAATCACCCATCCCCTGAGCCCT
CCTAACCCATTTGAAAAGCAAACTGGTTTCTGTATTAAATTCTTACGTGTTATTTATTATT
ATTATACAGATTTTGGTTGTCTATTGAAATTCAAATACTCTTCAAATGTATTTACTTCATTC
GTTTGTTGTTACGGCTAGGAAAAGTGCGTTACATAAAAGTAATGATAGAATATTAGTTTA
GATCAAGTCAGCCACGTTCATGGGCATCTCGTCGATTTGTGTGGAGTAGTACTGCTCAAT
GTCGCGCAGGATGCGGATATCGTCCGATTTGACAAAGTTAATTGCAACACCTTTGCGTCC
GAAACGACCAGAACGACCGATGCGATGGATGTACAGCTCACGGTTGTTGGGCAAATCGT
AGTTGATGACCAGCGACACCTGCTGTACATCGATACCCCGAGCCCACACATCGGTGGTGA
TGAGCACTCGCGACTGGCCGGCTCGGAACTCCTTCATGATCTCGTCACGCTCCTTGTAGG
GATAACAGGGTAATG

Gene: Dj1-a, Construct: pWhite-STAR_Dj1-a
LHA_F_Primer: AACAGGGTAATGGTACCTACCGATTAAATTACATATATGC
LHA_R_Primer: GCACTACGCGGCCGCATATCGGATGGGTACATTTCGTGGG
gBlock_Dj1-a_RHA:
CGGCCGCTATAACTAGTAACCCCCTTGTTATATCATTTGCAATTTTAGATCCTTGTTACCG
TGGCTGGTTTGCATGATTGTGAACCGGTGAAGTGCTCCCGATCTGTGGTCATCGTGCCGG
ATACTTCACTGGAAGAGGCCGTGACCAGAGGTGACTACGATGTGGTTGTTCTTCCTGGCG
GATTAGCTGGCAACAAGGCGTTGATGAACTCGTCTGCCGTTGGCGATGTGCTGCGTTGCC
AGGAATCAAAGGGCGGCTTGATTGCCGCCATTTGTGCCGCTCCCACGGCTCTCGCCAAGC
ACGGAATCGGCAAGGGGAAATCCATCACTTCGCACCCGGATATGAAGCCCCAGCTGAAG
GAACTTTATTGGTAGGTTTGCCAGTATTACGTGTTTATTAAATTATCAGGAAACTCTGACT
ACTCAGTTATATAGACGACAAGACTGTGGTCCAGGATGGCAACATAATTACAAGTCGTGG
TCCTGGCACTACTTTTGACTTCGCCTTGAAGATTACCGAGCAACTGGTCGGAGCTGAAGTT
GCCAAGGAGGTGGCCAAGGCAATGCTCTGGACTTATAAACCATGATGGGAATCGAAGGA

AAAGCTGATCATAAGTTACATAAAAATAAAAATATGAAACATTAACGTATAACGTATAGT
TATTTGCAAATAGTAAAATCTCTACTATTTTACAACCAATGATGCTGAAAAAATCGGTGT
ACTGATTTTTATGTACAAAGTTCCATAACTAGAATTTTCCTATATAGAATGACCTAGGTTA
TTGTCAATTAAAATATGAATATGTGGAAATTCATTTCGTATCTTTTTGATTTCCCTATTCAG
TAAACAACTGTACCTGTACCTATTTCCGCCATAATATTATCGAAGTTTCCCACCCAAATTT
TATATCAGAGTTTCCCATTTTGCCCGTTGGCGCTTTATGCACACCTTAGGTAAGACCGTAA
TACCGCCTAGTGAGACCATGCGAGGACTTAAGAATAACACCACCATACCGGGCGATTGC
AAATGTATTGAAATAAAACTAAAAGAAATCGTATCAACTGCTAGTTCGGCGAAACTCGG
GAAATCGGAAGGTTCAACTTTTGGGGCGGTCCGTGAATTTTTGCATTTTTCTGTGTTTTGG
CGGAAAACGAAACCGTTATTGAGATATTTGTGAATCGCAAACTGTAGATACAACTTTATA
GAGTAGTTACAGTCGGTAGGGATAACAGGGTAATG

Gene: Dj1-b, Construct: pWhite-STAR_Dj1-b
gBlock_DJ1-beta_LHA:
AACAGGGTAATGGTACCTACGATTGCTTTTCGTGCGTTTTCCGTGCAGTTTTCCTTGCTTTT
CCACACAGGCAATTGGAAATGCACACCAACACACACATGGGGCGCACACCAGCGCAGCC
ACACACGCACTGGCTTACACACACACACACTCGCACACATTCAACAGATATTGCATTTTC
TTTTTGGACTGGAAGATACGCAAATTCCAGCTGCAAACGTTGCATTTTCACCAGGCATTTT
CCGCAATTACCTTCTTCGCGTTTTCCACGTTTTCCACGCAATTTCCTCGCTTTTCGCCTCTT
TGTTCTCGCCTTTTTCGCGTTTCCCACACACGGCACTTATTGCTTTTCCTCTTTTTCTCCGTT
TTTCCTTGCCTTTTGACGACAATTCGCTGATATTTGCCGACGTATTTTCACTTGATTCACTC
GCGTTCGCAACCCATCACTTTTCGCGTGCATTCAATCGCATTTTTCAGGCATTTCCGATGC
GCGTTTGGCTTAAATTTAGTAAAATTAATTCGTGGTTTTGTTGCTGCCGTCGTGCTAGATA
TGTTCACATATCGATAGACCGATATCGATAGACAGAGATCTAGGGAGGAGCAGCTGTACT
TGGTCTGGCAACCACATCAGGTATCGATAGGATTTGACCGTGGTATATAAACATCGATAT
TATACTGAAAACAAATATAAAAATATTTCAGTGATCAGTTTATATAAATTTATGTAATAC
GACAGAATATAGAGAGCAAGTAAATGTTATCGGTTTTAACTTAAAGAACAAGACTTGTTA
GAATTGTTAATGAGGTTTCAAACCATTTCTGTGTAGCACGTAGCATAGACGCAATCCTTTT
CTCATATAAATAAAATCAACTGATTTCAAAGTCTTAATATTTTAAAATATAATGCAGAAC
ATTGTTTTGAGTAAGAATGAAAATATTAACTTCCTGTGAAAAACAGAAGTTTTAAATGCA
TCAAATTAGTCGCCTAACTGCAAGTCAAACCAAATTATACAAGTGTTTTAGTTTGCCGCTC
AGTTTTTGAAACCTCGGATATGCGGCCGCGTAGTGC
gBlock_DJ1-beta_RHA:
CGGCCGCTATAACTAGTAACATGATAGATCAAGGTCACCGTAGCCGGTTTGAATGGCGGG
GAAGCGGTGAAGTGCTCGCGGGATGTGCAGATCCTGCCGGACACCTCGCTGGCTCAAGTT
GCCTCGGATAAGTTCGATGTGGTGGTGCTGCCCGGCGGACTGGGTGGCTCCAATGCCATG
GGGGAGTCCTCGCTGGTCGGTGACTTGCTGCGCAGCCAGGAGTCTGGTGGCGGACTCATC
GCCGCCATCTGTGCCGCGCCCACCGTTTTGGCCAAGCACGGCGTCGCCTCCGGGAAATCC
CTCACCTCGTATCCCTCAATGAAGCCCCAGCTGGTGAATAACTATAGGTATACCACCTCTT
AAGTTTATTTTCCCATGATCTTCATCCCATTTCTTTTGATTATTTCTTTAAGCTATGTGGAC
GACAAGACGGTGGTCAAGGATGGCAATCTGATCACCAGTCGAGGTCCTGGCACCGCCTA
CGAGTTCGCCCTCAAAATCGCCGAGGAGCTGGCGGGCAAGGAGAAAGTCCAGGAGGTGG
CCAAGGGTCTTCTTGTGGCCTACAACTAACATATTTACACTTATTTATGAAAACAGATTGT
TTCAAGAGCAACTCATGCAAACAACAACTGAATTAAAATAAAATTATTCAAGACAGAAG
TTGTGATTCAATTTTATATTTGCTATTTGAAAGAAGTTTAAATGGAGAGCTAGGAAATGG
CATGTTGTTAAAGATATATTAAAGAAATTTATTTAAGTGAATTTAAATGTCATTATTTGAA
GCAAACGGTTTTCATTTAATTAGTTTTGAGCGAAGTATAATTCTATCATAATGAATCAACT
GAACTGCGTTAAAATAAATTGTTCAATGGGCTTTAATTTAATTTGTCATGAAATATAAATG
CATATATGGCATGAAAAAAGAAACATCTATATTATTTCATTTGGTTATGACTGGACTATA
AATGAAATTAAAAATGCATATATTAAAATGGGATGTGAAGCCAAATGGAAGTATTGCTG
ACGATATATTTACAAAATAGTACATTATTAATATATTTAGTTATATTCAATATTTACATTG
ATTAAACATTCTGCTGCGTAGGGATAACAGGGTAATG

Gene: Rme-8, Construct: pWhite-STAR_Rme-8
LHA_F_Primer: AACAGGGTAATGGTACCTACAATGGTAGTTAAGTTCAG

LHA_R_Primer: GCACTACGCGGCCGCATATCCAATTCATGACTGGTCTT
RHA_F_Primer: CGGCCGCTATAACTAGTAACTTATAAGATAATACGGAGAGCTCGGAACT
RHA_R_Primer: CATTACCCTGTTATCCCTACAGGCAGAGTATACGCTTC

Gene: auxilin, Construct: pWhite-STAR_auxilin
gBlock_Auxilin_LHA:
AACAGGGTAATGGTACCTACCGGTGGTAATTAGTACACTGATAAGGTTGTGTATTTTGCA
TTCGACAAATCTCCACGCGTTACGTGAAACTGGCCAGGTGCACACAGCAAATTGCAAGCT
TAACTTCAATGTCCAAAATAAGCGGAGCTCGGTTTGCGCAAACTTACGGCCATATCGGTC
ATCTGTAACTATCCGTATTCGCCAGCGGCCCAAGCGTAGCGCAACACAGCTCTCAATTAT
CCAAAGACAGATGTGTAAATATACTGCCGTTACCAACTTGTTGCTGTTCCACGGAGGGGA
GGTGCCCCAAAGGCTTACTAGTCATACTCCATTTCTTCATTCCGACGGCCAGCTGTTGCAG
CGGTTGTCCCCTGGGTTTCCCCTTAGCTGTTGACTTCAATCAGCCAACTCTACCTGGCACA
ATGTACATAGGAAACTCAAACTTCAGAGCTGGACCTTGAATTGGCTCAAAGACACATGCA
CGCTTGATGCACAGCTAAATGCGGGAATTTACTCAGGCGACTCAAAGGGGAATGACTTTT
CGAAAAAAGTGTTTAACCTTTCAATTACATTTTATTTTCTTTCAAAGCTGTCACAGCCCCA
GTCAAAAATGCAATACCTCTAGACCTCTGATAAATGTGGTTGTGTGCGCTCTTTTAACCGT
ATCAAGCTAAATGGGTACTTGTTGAAGAGTATGAACTAGCGGAAGGAACTCTTTAGAGCA
TATATCTTGACATGACTGCTCGTACAAAAACTGTGATCTCTTAAAACCTCGCAAGGGCGT
TGGCACTGCACGCATTTTAAATATAATACAATCACCTCAGGTGCAATCTTAAAGCCAGGA
TTAAACGTCTGAATTTTGAAGAGTCAACATACAAAATGTTTGCTTAAAAGCACTCTAAAT
TTAGTCGAAATTCATAATCTAGTTATCCGCGCGCCACATCGATAAGAGCCCAGCGTCAAA
TATCGATAAGAGCAGATATGCGGCCGCGTAGTGC
gBlock_Auxilin_RHA:
CGGCCGCTATAACTAGTAACTCGAGAGGCGCAGCTCTGCTTGAGCCAAGCCAACGGACG
AATTGTGGCGATAGTGCATTGCCACCATCACGGTTACGCGTTACCGCTGGTGCCCACCTG
CGAGCCATGGCACATTAAACCGATCGGGTTTTTTTTAACAAGTTCTTATATAAGAACAA
CAGTGGCCTGAAGGTGGCTGAAAGTTCGTAGCTCTGCAGGGAGCTGGAAGACAGAATTC
GTAAATATATAGCAGGCTACCAGCCGGTCAACCCCTTCCTCCGAGCGTGCCGCACTTAGC
ACTTAGGCAGGGCCTTATCTGTGCCTCGTTATCTACTTGTCTCTCGCTTCCACCCCCTTTTT
CAGGCGGATACGCCTTCGTGTATGTAGCCCAAGATGTGCAAACTGGCACAGAATACGCCC
TCAAGCGTCTGATCGGGGCTGACATGCAAGCCTCCACCGCCATCATCAACGAGATCAACA
TCCACAAGCAGCTGTCGGGGCACGAGAACATTGTCGCCTTTGTGGGTTCCAGTTATACCG
CCCCATCAACTCAATTGGGAGCCCAGTACTTGCTCCTCACCGAGCTGTGTAAAGGTAAGG
GACGCCCTAGGGCGTCCAATCATACTACGTGAACATTTGAGCACATGGATTTCTATCCTT
GTCAGGCGGATCTCTGGTGGACTGCTTCAGAACAAACAATGCTCCATTCAATCCGACTTG
TGTTCTGCGCATCTTCTACCAAATGGCGCGGGCTGTGGCCAGCTTGCACTCACAGTCACC
GCCGATAGCCCACCGAGATATAAAGGTTGGTCTCCAACCATAGCTGGAAGTAACAGACT
AATACCCTAATCCTATTTGATTGCAGATTGAGAACTTTCTCATTGGCAACGACAAACAGA
TCAAGCTGTGCGACTTTGGGTCCGCCAGCACGGAGGTCCTGTCGCCCACGTTTGAGTGGA
GCGCCAACCAGCGCGTAGGGATAACAGGGTAATG

Gene: eIF4G, Construct: pWhite-STAR_eIF4G
LHA_F_Primer: AACAGGGTAATGGTACCTACATGGCCGCTACGCATCTT
LHA_R_Primer: GCACTACGCGGCCGCATATCGTTATTTCACATTTTTAAACGCTT
RHA_F_Primer: CGGCCGCTATAACTAGTAACCATTCAGCACAAAATATG
RHA_R_Primer: CATTACCCTGTTATCCCTACATATTTATTTACTCGGC

Gene: nutcracker, Construct: pWhite-STAR2_nutcracker
LHA_F_Primer: GTCTCTAATTGAATTAGATCCCTGTAAATATGTTTAAATCT
LHA_R_Primer: CGGCCGCTATAACTAGTAACACTATTTTAAACAAGTTGGAACTGA
RHA_F_Primer: GGCACTACGCGGCCGCATATCTAAAAAGAATATTCCTTGCTTTATT
RHA_R_Primer: TAACAGGGTAATGGTACCTACCGAGCAGCAAGCATCCCGC

Gene: Gba1a_1b, Construct: pWhite-STAR2_ Gba1a_1b
LHA_F_Primer: TCTCTAATTGAATTAGATCCCAATGAAATGATGTTCAACA
LHA_R_Primer: GCGGCCGCTATAACTAGTAACCTATTATCTTACTTCTGTCCAGTCG
RHA_F_Primer:
GGCACTACGCGGCCGCATATCAGAAAACAATAAATTCATGCCTAAATGGCCATGAGACTC
CTCAA
RHA_R_Primer: TAACAGGGTAATGGTACCTACTGCAGATCCCGCGGATCGA

Gene: punch, Construct: pWhite-STAR_punch
LHA_F_Primer: AACAGGGTAATGGTACCTACGTTTGAGGGCAAAAGCTT
LHA_R_Primer: GCACTACGCGGCCGCATATCTCATTGACTAACTAAATC
RHA_F_Primer: CGGCCGCTATAACTAGTAACACTGATCAAGACGCCGGA
RHA_R_Primer: CATTACCCTGTTATCCCTACTTGGGGGGAGGTTGACTT

Gene: Gdh, Construct: pWhite-STAR2_Gdh
LHA_F_Primer: TCTCTAATTGAATTAGATCCCGGGGTCCATTCAAAACCGA
LHA_R_Primer:
CGGCCGCTATAACTAGTAACAGACGAAATGCCAGAGTAACAGCAACAACCGGCTTGATC
AGTC
RHA_F_Primer:
GGCACTACGCGGCCGCATATCTAATAATAATATCTACATTCTTGGGCACCACTCCCGGCT
GGG
RHA_R_Primer: TAACAGGGTAATGGTACCTACATGATACTTCAAAATTATA

Gene: Omi, Construct: pWhite-STAR_Omi
LHA_F_Primer: AACAGGGTAATGGTACCTACAGAGGAGCGTCGAGCTAA
LHA_R_Primer:
GCACTACGCGGCCGCATATCAGACCACATTCGGAAGTATGTTTTTAGCTATTCAGCTATA
CGTTttAACTAGAAACGACAGCA
RHA_F_Primer: CGGCCGCTATAACTAGTAACACGTAAATCAGACATCCGATCTGG
RHA_R_Primer: CATTACCCTGTTATCCCTACCGCACCCGATTAGGAATA

Gene: Lrrk, Construct: pWhite-STAR_Lrrk
gBlock_Lrrk_LHA:
AACAGGGTAATGGTACCTACTTGGATATCTCGTGAAGTACACAAACCAAAACAATGAGC
GACTCCGACGAAGGTGAGATAACACAACTTTTAGTGAAATTATGCACAATATGCTAGTTT
TGCGCAAATTTAGTCAGAGTGCAAATGAGCATTATACGCTTGACTATGCAAATTACCTAA
GTGGTGCAATTAAAACTTCAACCAGTTGTTATTGGTTTTTCAACAACACCAGCAAGGGCA
CAGGGTGACATTGGTGCAATGGAAGTGGGCGCGCAAAAACTTTTTTCAAACAGTCATCCC
CCACAGAAGCAACTGGTGCAACCACTGGACCATCCGACACCCACGAAGAGACAAATCAA
AACAAATGCCACGCACTCACCCAATGCCACATTCTTGAACAAGTGCAACAGTTGCCGCGC
TTCTTGGGCTCCGACTTGCGCAGTGCGACATAAAACGGCCTGGATCAATGACCACAAAAA
CGTGGCCATCGATTTTCCGCAGTTCAGTTTTTGCTAGCGGCGCGTTCGGTCATCATACCGA
TGCCAATTAATTTTTGGGGCTTATTCGCACCGATTTGGGTTACATAATGTCATGGAAATTC
AGCATGGAACATCCCAAAACTGGAACTGAAACAGCTCTGGAAGCCTGTGACTACTTCGTG
GATGAGGTCATTGAGGCATCCTCAATACGGGGTGGGAATTGGTTTATTCCAAAGGTGCTG
TGGATATGCGGCCGCGTAGTGC
gBlock_Lrrk_RHA:
CGGCCGCTATAACTAGTAACGTTTTAGAGCTTGTCAGTCTGGTTACGAGAGCATTACACA
GCGATTGCTGGATGCTGGAGCGGATGGTCGCTCCCATGCCGTGACCAAGTACTCACCCTT
GTACGCCGCCGTCCATAGCGGTCACTTGGGCATTGCCCGACTGATGCTAGACCATTTTCC
AGAACTGATCCAGCAGCCGACTGTAGAGCGCTGGCTGCCGCTGCACGCCGCCTGCATCAA
TGGACACATCAAGCTGCTGGAACTCCTTATCAGCTACAGTTATCCCGACTACCTCTACCA
GACATATCGCGACGAGGAGGGCCAGTGGGAATGGCGGCTCCCCTTCGACGCTAACGCTC

ATGATGTGACGGGTCAGACGAGTCTGTATATTGCCAGCATCCTAGGAAACAAGCAGCTGG
TTGGTGTGCTTCTAAAGTGGCAGCTCCATTGCCGGCGTACGTTGGGCGATTCCGCCAGCTC
GGTGAGCACTCCTATTACGCCCACCAGGAAACGTATTTCATTTGGCATTCAGGCTATCAT
GTCCAAACTGCACATATCTGGAGAGTCAGAAGGACCCGACGACCTAGCTTCACAAGAGT
CAACCGAGTGCCAACGGTGTCCCATTAACGTGAATCTGCTATGTGGAGCGGCGAGAGAA
ACAGCTCTGCTTGCGGCCGTTCGAGGCGGCCACCTAGACGTGGTGCAGTCTCTGCTACAG
CACGGCGCAAATCCGAATATTGTAGCCAAGCCAGTTGAGGATCACAACGACCCGAAATG
TTGCGAGGAAATATATGGGCTCAGTAATGTCCCCATTGCAGAGGCCTGCAAGCAAAGGTC
GCTGGCTATGTTGGATCTCTTGCTTAAGCATGGAGCCCGCGACGATAATGGCACGGCCAT
TGGAATGGCTATTACATGCGGCGACGAGGCCATCCTGAGTCGTCTTTTGGCCCGACGAGT
CCATCCGGACTCAGACTACAAGATCAACAAGAAGGGTCTTCCCACACCAGTGGAGGTGA
ACGTGTTTTTGCCGTCCACCAGCAACGTAGGGATAACAGGGTAATG

Gene: Tango14, Construct: pWhite-STAR2_Tango14
gBlock_Tango14_LHA:
TCTCTAATTGAATTAGATCCCGAATCCGGCGCATGGTCACACTGACGAACACGTATTTTC
ATTATTCTTGCGAAGCGCATCGCTTAAAACTTGATTACGCTCGGATAACAAGATGCACCC
GCGTCTCAAGTGACTGAACAGGTAGCGAAGTGACCCTGGATGAGCGTGGGTAGGCAGCC
GGCACCTCTGGAAAGCCCTAATCCCAGGTCCGCCAGCCGTTTGCAGGTTATGTCCGCTTG
TGTACACTGTGACGGGGAACCGAAGAAGCAACAGGAGCGGATTCCTGGCAGAAGAGACA
ACCGCTGTACGAGATACCGCCCCTGACCTGCGCAACATGATCGAGGTGCTGTGCCTGCTG
CTGGGCAGGATCCTGCTGCTCCTGGTCGGCGGCTACGAACTGATGTGGCGGCTCCGAGAG
CGACTGAATGCACTGGCCGTCCGGTCATATGACCTTTGGCGCAGCAAAGCGGCACGTGAG
GCGCACGAGCGGCGCGTGCTCGCTGACTGCCGCTCCCAGTTGACGAAGACGCCGCAGCAT
CTGGTCCTGGTTATCTCCCCCGTAGATGCCGGCGTGGATGCGGTGCTCCTCAGCAGGATCT
TCGACTTTGCCCTGGACGTGGGCATCAAACACGTCAGCCTGTACGACAGGCGGACGAAA
GGCAGGGGATACGTGGACATGGCCGATCTCTGTCGATCCACCAACGCGGACACGGGCAG
CTGCTTAAAGTGGCCACCCGTAGCCAGTCCCAGCAAACTGGAGAACCAGCCCAAAAACG
GACAAAAGACAAATGGTTATGTGAACGGTTCACATTCCCCTCAACTGCAGGTGAGTGGCG
GCGTCTGATAAGCCGTGATTAGTAATGGACTTTAGAACGATGTCGGAAAATGTCTTACAA
GTATTACACTTTTTGTTTCAATTGTTAAATAAAAGAAGTAATGAGCTGGAGATTTAATTTA
GTTCATCGTTACTAGTTATAGCGGCCGC

RHA_F_Primer: GGCACTACGCGGCCGCATATCGTAGCGACACCCTTATATT
RHA_R_Primer: TAACAGGGTAATGGTACCTACCCTCGACTAACGAGAGCAA

Gene: Pink1, Construct: pWhite-STAR_Pink1
gBlock_Pink1_LHA:
AACAGGGTAATGGTACCTACGGCGGCTCCAACGGGCACTTCGAGCTAAATGTATTCAAAC
CCCTGGTCGTGTCCAATGTGCTGCGCTCCATTCGCTTGTTGGGTGAGTCGGAAATGTGTTT
TCCAAGTGGAAAAACATGCATACATAACCTTCTCCTCCCAGCTGATGGCAGCATGACCTT
CAGCAAGAACTGCGTGGAGGGACTGCAGGCCAACAAGGAGAGGATCGACAAGATCATG
AACGAGTCCTTGATGCTGGTGACCGCGCTGAATCCGCACATTGGCTATGACAAGGCCGCC
CTGATCGCCAAGACGGCGCACAAGAATAAGACGACCTTGAAGGAGGAGGCACTGAAGAC
CGGAATTACCGAAGAGCAGTTCAAGGAGTGGGTCAATCCCAAGGAGATGCTGGGACCGA
AGTGATTCGCTGTGCGGCTATTGACGATTTTGTTTTAGTTTCGTGTAATAAATTTTTGTGTG
TAGCAGGATATTTCAATAGATTGTATTAATTTGTTGATTTTACGGCAGGTGAATAACGGTT
TTTAGTTTTTGAATTCTCCAAATGATTCGAGTCCACACTACGCGATAGTCATTATCGATTC
TAAAGATATACCGTAAGTGTGGTCACACTAACGCCTTGCTGTTTCTAAATTATTCCGTTGT
TTTTATCGTAAATTCTTGCACACTTAGCTATTATTTATAGCGAAATATAAGCAAAGCAAG
CTTTTAAACAAGTGTTTATTGCGGCGCACATTTTCCGTGACAAATGAAAAGATCCAGCTG
ACCAGCGATTATCTAACGACCAAATCGAATCGAAACGAAACGTTGAAGTAGGCGCATTA
CATTAAAAATTGGCTGCCAGGCAAGTTGGAGGTCGTAAATTAATCGTTTTGTTCAGTTGA
AAATAGCCCAAAATCAGTCTAGCCCACACAGATATCGAAAAAGGCGATATATCTTTGGAT
AATCATTGCAAAAATAAACCCTTTCGATTCGACGATTTCGCCTTGTAGTTCTGCCACCACC

GCCCCCtCAaTTCGCCCCGCAGCTGCACCACAACCACCACCACCCACAAACAGAAAATTTA
TTTGCGACGCAAAAACAAAACAATCGAATCGAAACGAACCGAACCGAAACGAAACGACA
GCAGCTGTTTGGTTATAAATCGGCGCTATCGATCGCACACATGAGCAAAATTGGCGGAGA
AGAAGAGGAAGCAGCAGCAAGAGTTAAGCACCACAAATCTTAAAGAATAGGTAAGATAT
GCGGCCGCGTAGTGC
gBlock_Pink1_LHA:
CGGCCGCTATAACTAGTAACCATCCAATCTCTTCGCCAGACAACGCCCCAGCAGGCGGCC
AAATCGGTGGTCAATGTAGTGCCCCGCACCATCAACTCTCCGTCGGGATCACCGTTTAAT
GGCAGCGGCAGTAGCCCCACCAGCAGCAGTGGAATCTTCCGAGTGGGCCAGCATGCGCG
CAAATTGTTCATCGACAACATCCTCAGCCGGGTGACCACCACCTACTCGGAGGATCTGCG
CCAGCGCGCTACCCGCAAGCTATTCTTTGGCGATTCAGCGCCCTTCTTCGCCCTGATTGGC
GTTAGTCTGGCCTCCGGATCGGGTGTGCTCAGCAAGGAGGATGAACTGGAGGGCGTGTGC
TGGGAGATTCGGGAGGCAGCTAGCCGGCTGCAGAACGCCTGGAATCACGACGAGATCTC
CGATACGCTAGACAGCAAGTTCACCATCGATGACTTGGAAATCGGTCCGCCCATAGCCAA
AGGTTGTGCCGCTGTCGTCTATGCAGCGGATTTCAAGAAAGATGTTGCCTCGGATGGTGC
ATCCCTGCATACCGATGCGCAGCCGCAGGCAACGCCAGCCTTTGCGCCGAATAGCTGGAG
TACCCACGAGATGATGTCGCCGCTGCAGAACATGTCGCGCTTTGTTCACAACTTTGGCGG
CTCTGTGGACAACGTCTTCCACTACAGCCAGCCATCTGCGGCCAGTGATTTCGTGGGCGC
CCAGTCGAGGGAACAGGACCAGCGGCACCACGAGCAACAGCAGCATCAGAATCAGGAA
CAAGAGCAGCATCAGAACCAGGAGCCCAGCAGCAGTGCCTTCAATGTGGTAAATATGTA
AGCTTAAATCTGCATGGCAACTTGCCATCACGCTCTTTGCTTTAACTTGCAGACTTCTCCA
GCGAATTCAAACATCAACAGCTCTGTGGACAGTTATCCACTGGCACTCAAAATGATGTTC
AACTACGACATCCAGAGCAACGCTCTGTCCATACTGCGTGCCATGTACAAGGAGACGGTA
CCGGCACGCCAGCGCGGCATGAATGAGGCCGCCGATGAGTGGGAACGTTTGCTGCAGAA
TCAAACTGTTCACCTGCCGCGGCGTAGGGATAACAGGGTAATG

Gene: iPLA2-VIA, Construct: pWhite-STAR2_iPLA2-VIA
LHA_F_Primer: GTCTCTAATTGAATTAGATCCCGGCTCGAGGAGCTGGACTC
LHA_R_Primer: CGGCCGCTATAACTAGTAACCCAATGGTAGGGGCTTGCA
RHA_F_Primer:
GGCACTACGCGGCCGCATATCTACTACAATGTAAATGGGCTGCAGAAAGCTTGCGATGCC
TTGGCTGATAATCCCTCCTAAACGCTGTCCCATTTGATTG
RHA_R_Primer: TAACAGGGTAATGGTACCTACTCCGGTGCCTCTGGCGGAA

Gene: Loqs, Construct: pWhite-STAR_Loqs
LHA_F_Primer: AACAGGGTAATGGTACCTACGTGGCGTCTAGCAAGAACA
LHA_R_Primer: GCACTACGCGGCCGCATATCATGATCGTAAGTTTGTTTA
RHA_F_Primer: CGGCCGCTATAACTAGTAACGACTGGGCTCACGGTCGCC
RHA_R_Primer: CATTACCCTGTTATCCCTACATTTGAAATCACACACGCC

Gene: Rab39, Construct: pWhite-STAR_Rab39
LHA_F_Primer: CGGCCGCTATAACTAGTAACAGACAACAAATTCGCCGAGGTGCG
LHA_R_Primer: CATTACCCTGTTATCCCTACTGAATCAACAACATGATG
RHA_F_Primer: AACAGGGTAATGGTACCTACAATCCAAAATAATTAATAA
RHA_R_Primer: GCACTACGCGGCCGCATATCAAAGTAATCGATAGTAGC

Gene: Synj, Construct: pWhite-STAR_Synj
gBlock_Synj_LHA:
AACAGGGTAATGGTACCTACCAGAATTTTGCAATAAAATCGCAAACAAAGGTATTAGGA
GTATTAAGAGGCGTAAAAAATTCATTTGAGGAGCCAATAGTTTCGTGAAATCTAGTGTTT
CCTCTTTTAAGTGGTCGCCAAGCATGCCGTTCGTTGGCTGCCTCTAAAAACAGTCAAATGC
CTGTGCAAATTTCTCGTGAAAAATCCATAGAATGCACAAAAAATGTTTATCAGTCACTCA
CAAACACACGCACGCGCCAGCAGTGAGTGGGAGAGAGCGAGAGAGAGCACAGCTGCCG
CCGGTGCATGTCATTGCTGTGTGTGCGTGTGTGGGTGAACTTGAGTGGGAGGCGGAAGAT
ATTGGAAAAATTCCTCCGCTTTTCGGTTTGTTTATGTTTAAGAGTTGCCTATTGATTTTTAA

CAGGCGCCCGAAATTTTAGTGCCATTTCGGTTCAAAGTGTCTGTTGTACTAGTTGAACTTT
CCCATTTCCACTCACCTCGTGCAAATCCTAAAATTAAAAGAATCAAAAACTTTGTGGTATT
AAATTAACCCCAAATTTGAGCTCATTTGAGCTATTACGAAGTGCACAAAATAACTTAACT
TAGCAAGTAAAACACTCACCAAAATATTTTTTAAAACTTAAAAAAACAACGAATGTAGCG
TAGTTGATAATAACTTATTACTTAGTAAAAAAAATATTACGATAAATTAGTTTTAAAAGT
GTTGCTCATAAATTGAAATCGAATGAGTCCTATAATTGTAGCATTGCAAACGACGAATCC
CAATATTAAACCTACACCTTAAAAACGAAGGATATGCGGCCGCGTAGTGC
gBlock_Synj_LHA:
CGGCCGCTATAACTAGTAACCATATTTAGGAAGCCATTGATGTCCTGCTAGTGGGCTCCA
CGCTTAGCTCGGAGCTTGCGGATCGGGCTCGCATCCTACTGCCCTCCAATATGTTGCATGC
ACCTACCACTGTGTTGAGAGAGCTATGCAAGCGCTACACTGAATATGTGCGTCCTCGAAT
GGCACGTGTAGCCGTGGGTACCTATAACGTCAACGGCGGCAAGCACTTCCGCAGCATTGT
GTTCAAGGATTCGCTGGCCGATTGGCTGCTCGACTGCCATGCCCTTGCCCGCTCCAAGGC
GCTTGTAGATGTGAACAATCCGTCGGAGAACGTCGATCATCCGGTGGATATCTACGCCAT
TGGATTCGAGGAGATTGTGGATCTGAATGCTTCCAACATAATGGCGGCCAGCACCGACAA
TGCCAAGTTGTGGGCCGAGGAGCTGCAGAAAACGATCTCGCGGGACAATGACTACGTGC
TGCTCACATACCAGCAACTGGTGGGCGTGTGCCTATACATCTACATCCGACCGGAGCACG
CGCCGCACATCCGGGACGTGGCCATCGACTGTGTTAAGACAGGATTGGGTGGTGCCACTG
GGAATAAGGGTGCCTGTGCCATTCGATTTGTGCTTCATGGTACTTCCATGTGCTTCGTGTG
TGCCCACTTTGCAGCCGGACAGTCACAGGTGGCTGAAAGGAACGCTGACTACGCGGAAA
TCACCCGGAAGCTGGCCTTCCCGATGGGCAGGACGCTAAAATCACACGACTGGGTGTTTT
GGTGCGGCGACTTCAACTATCGCATCGACATGGAGAAGGACGAATTAAAGGAGTGCGTA
CGTAATGGAGATCTCTCAACCGTCCTCGAGTTCGATCAATTGCGCAAGGAGCAGGAGGCT
GGCAATGTGTTTGGCGAATTCCTCGAGGGAGAGATCACTTTCGACCCGACGTACAAGTAT
GATTTGTTCAGCGACGACTAGTAGGGATAACAGGGTAATG

Gene: Vps13, Construct: pWhite-STAR2_Vps13
LHA_F_Primer: TCTCTAATTGAATTAGATCCCCGCCTGGATGTGGTTGTTC
LHA_R_Primer: GCGGCCGCTATAACTAGTAACGAAACCAAACAGATGGAAC
RHA_F_Primer:
GGCACTACGCGGCCGCATATCGTGAGTTCCAGTTCCCCGGAGAGGATGACACCATTCTGC
TCC
RHA_R_Primer: TAACAGGGTAATGGTACCTACTGATAACAGTGTTGTAACA

Gene: Vps35, Construct: pWhite-STAR_Vps35
LHA_F_Primer: AACAGGGTAATGGTACCTACCACGCAATATCCACCACA
LHA_R_Primer: GCACTACGCGGCCGCATATCCTGTTTCTTAAGTTTTTTGAACGA
RHA_F_Primer: CGGCCGCTATAACTAGTAACAAAATTAAAGCGTCTTTATACAAC
RHA_R_Primer: CATTACCCTGTTATCCCTACCTGAGCCAAGCTCAGCAG

Gene: VAC14, Construct: pWhite-STAR2_VAC14
LHA_F_Primer: GTCTCTAATTGAATTAGATCCCCCGTGGAGCAGGCGCCCGC
LHA_R_Primer: GCGGCCGCTATAACTAGTAACTGCTACTCACTTCTCGATC
RHA_F_Primer:
GGCACTACGCGGCCGCATATCTATTTGTGACCATTAGTCATCAAAATCGTGTTCCCATTTC
AA
RHA_R_Primer: TAACAGGGTAATGGTACCTACCTGTGAAAGAACGTCCATA

Primers used to verify the correct integration of the IMCE knock-out cassette

Knock-ins made with pWhite-STAR used the combination of the F_LHA_junc_primer with R_w_start_primer and R_RHA_junc_primer with F_w_end_primer for verifying a correct cassette integration.

Knock-ins made with pWhite-STAR(II) used the combination of the F_LHA_junc_primer with F_w_end_primer and R_RHA_junc_primer with R_w_start_primer for verifying a correct cassette integration. This distinction is due to the reversed orientation of the mini_white cassette in the pWhite-STAR(II) construct.

F_w_end_primer: TTCGGAGTGATTAGCGTT
R_w_start_primer: GAGTGAGAGGTAATCGAA

Gene	*F_LHA_junc_primer*	*R_RHA_junc_primer*
ATP13A2	GAGAGCAAAGGAAAAGGAA	CTAGTCTTTAGCCGCTTTT
Vham8.9	TGTATATGTCTGGTGTAGG	TTGTAGGGATTGGTGGTG
CHCHD2	GAAACATCGTCCCTTTTGTA	TCTTGTGGAGGATGTGGT
Coq2	AGTCGGACGAGATATTTG	GAGCGCGAGGAATGGAAA
Dj1-a	CAGAAAGGAATCGCATTG	CTGTTGCTTTGGTTGTTG
Dj1-b	GAAAGAGAGGGAAGACAGAA	CCCACTACAACTACAACA
Rme-8	CCGTCCGTCTTTATCTTTA	GTCGTTGGTGCTGTAGTT
auxilin	CACTGCACCCGGAAAATCA	GGTAGCACAGGAAGTAGAG
eIF4G1	GAATTGAACGCTGGCATA	TTCCCCATTTTTTTCGCC
nutcracker	TTTAGTTACATTAGGGCTGG	GGGAAACCGAAAACATAG
Gba1a_1b	GCGTTATTTTGCCCACAT	ACTTGAGGTGATAGAGGG
punch	GTGGGTTTCAAGGATATG	TGTGAGTGCTAAAGTGGT
Gdh	TTTCGTTTGGTTCCCTGT	TTAAGGATGGATAGGTGG
Omi	GAAGAAACACGATTTGGGA	AGACAGACGGATGGTAGG
Lrrk	TCGATATAGGTTACAGTTGG	TATTCGCGTGATAGCTGT
Tango14	GTGCAATAGCCAAGCTTA	AAGTCAGGTGAGTGTAGAA
Pink1	GGTCTGGGCGAACTAATG	CACCTCGTCGCAAAAGAA
iPLA2-VIA	CTGCTGGACAATGGGTAT	TTTACATACTGTGCCGGTT
Loqs	GAGAAACAGGGCTTTAAG	GCCCGTTGCATTTTTTAG
Rab39	TATAGGAAAACACTCGGG	GGAGTACTGGTGGGTCATA
Synj	CATATAGAATGCTGTCGT	CTGTTTCTCCGAGGTGTC
Vps35	AAGGCGTTGTTGTAGAAG	AAGGCGTTGTTGTAGAAG
VAC14	TGTGCCCCAGATAGAAGT	TGTGCCCCAGATAGAAGT
Vps13	TGTTTTGATGTGCTGGTATG	TTAACGACCAAGCGACCC

References

1. Pech, U. *et al.* Synaptic deregulation of cholinergic projection neurons causes olfactory dysfunction across five fly Parkinsonism models. *eLife* **13**, RP98348 (2025).
2. Li, H. *et al.* Fly Cell Atlas: A single-nucleus transcriptomic atlas of the adult fruit fly. *Science* **375**, eabk2432 (2022).
3. Verstreken, P. *Supplemental Figure 12a Schematic of Experimental Approach: Q10 and R55 Treatment.* (2026).
4. Guo, J. *et al.* Coding mutations in *NUS1* contribute to Parkinson's disease. *Proc. Natl. Acad. Sci. U.S.A.* **115**, 11567–11572 (2018).
5. Pesah, Y. *et al. Drosophila parkin* mutants have decreased mass and cell size and increased sensitivity to oxygen radical stress. *Development* **131**, 2183–2194 (2004).
