# Supplementary material for: Behavioral screening defines the molecular Parkinsonism-related subgroups in Drosophila
Source: Nat Commun. 2026 Mar 10;17:3761. doi: 10.1038/s41467-026-70303-8 (PMC13106710; doi:10.1038/s41467-026-70303-8)
Supplement: Supplementary file 2 — Description of Additional Supplementary Files [file 41467_2026_70303_MOESM2_ESM.pdf]

Description of Additional Supplementary Files

Supplementary Data 1:

Normalized depolarisation of single heterozygous mutants, average Genetic Interaction strength of all gene pairs including Bayesian estimate of interaction significance, related to Fig.3. (1. tab: single_het_gene_norm_depol) “single_het_gene_norm_depol” relates to the depolarisation amplitudes of single heterozygous mutants normalized to controls of the same experimental recording. (2. tab: Bayesian_statistics) “Observed” relates to the mean normalized depolarisation amplitude of the gene pairs. “Expected” relates to the expected normalized depolarization according to the non-interacting model based on single heterozygous mutants, while the “mean GI strength” is the subtraction of the modelled expected depolarization from the individual observed depolarization amplitude of heterozygous gene pairs. Hdi0 and hdi1 delineate the limits of the 95% high-density interval (HDI). Dist_0 show the distance from the HDI to zero. If dist_0 = 0 the interval contains zero.

Supplementary Movie 1:

Control, related to Fig.1. Seizure-like behavior of control flies at 26±2d. Scale bar: 1cm.

Supplementary Movie 2:

DJ-1abKO-WS, related to Fig.1. Seizure-like behavior of DJ1a/bKO-WS flies at 26±2d. Scale bar: 1cm.

Supplementary Movie 3:

iPLA2-VIAKOWS, related to Fig.1. Seizure-like behavior of iPLA2-VIAKO-WS flies at 26±2d. Scale bar: 1cm.

Supplementary Movie 4:

Pink1KO-WSy, related to Fig.1. Seizure-like behavior of Pink1KO-WS/y flies at 26±2d. Scale bar: 1cm.

Supplementary Movie 5:

Rab39KO-WSy, related to Fig.1. Seizure-like behavior of Rab39KO-WS/y flies at 26±2d. Scale bar: 1cm.

Supplementary Movie 6:

Rme-8KO-WS+, related to Fig.1. Seizure-like behavior of Rme8 KO-WS/+ flies at 26±2d. Scale bar: 1cm
