# Supplementary material for: Behavioral screening defines the molecular Parkinsonism-related subgroups in Drosophila
Source: Nat Commun. 2026 Mar 10;17:3761. doi: 10.1038/s41467-026-70303-8 (PMC13106710; doi:10.1038/s41467-026-70303-8)
Supplement: Supplementary file 10 — Reporting Summary [file 41467_2026_70303_MOESM10_ESM.pdf]

Corresponding author(s): Patrik Verstreken

Last updated by author(s): Dec 28, 2025

Reporting Summary

Nature Portfolio wishes to improve the reproducibility of the work that we publish. This form provides structure for consistency and transparency in reporting. For further information on Nature Portfolio policies, see our Editorial Policies and the Editorial Policy Checklist.

Statistics

For all statistical analyses, confirm that the following items are present in the figure legend, table legend, main text, or Methods section.

n/a	Confirmed	
☐	☒	The exact sample size (*n*) for each experimental group/condition, given as a discrete number and unit of measurement
☐	☒	A statement on whether measurements were taken from distinct samples or whether the same sample was measured repeatedly
☐	☒	The statistical test(s) used AND whether they are one- or two-sided<br>*Only common tests should be described solely by name; describe more complex techniques in the Methods section.*
☒	☐	A description of all covariates tested
☐	☒	A description of any assumptions or corrections, such as tests of normality and adjustment for multiple comparisons
☐	☒	A full description of the statistical parameters including central tendency (e.g. means) or other basic estimates (e.g. regression coefficient) AND variation (e.g. standard deviation) or associated estimates of uncertainty (e.g. confidence intervals)
☐	☒	For null hypothesis testing, the test statistic (e.g. *F*, *t*, *r*) with confidence intervals, effect sizes, degrees of freedom and *P* value noted<br>*Give P values as exact values whenever suitable.*
☐	☒	For Bayesian analysis, information on the choice of priors and Markov chain Monte Carlo settings
☐	☒	For hierarchical and complex designs, identification of the appropriate level for tests and full reporting of outcomes
☒	☐	Estimates of effect sizes (e.g. Cohen's *d*, Pearson's *r*), indicating how they were calculated

Our web collection on statistics for biologists contains articles on many of the points above.

Software and code

Policy information about availability of computer code

Data collection	To collect sleep behavior data ethoscopes were used (Geissmann et al., 2017). Confocal images were acquired using Nikon A1R confocal microscope. Details can be found in the Methods section of the manuscript.
Data analysis	All code uses open source packages (R v3.6.3 and R v4.2.3, Scikit-Learn version 1.0.1 for Python 3.9.6, PyMC package version 5.10.4 for Python 3.10.14), which are described with detailed version number in the Methods section and are deposited on Github and zenodo (https://github.com/verstrekenlab/drosophila-parkinsonism-subgroups, DOI: 10.5281/ZENODO.18032710). Quantitative imaging data were analyzed using Fiji (ImageJ 1.53c).<br>GraphPad Prism 10.1.2 (San Diego, USA) was used to determine all other statistical significances. Datasets were tested for normal distribution using the D'Agostino-Person Omnibus and Shapiro-Wilk normality tests. For non-normally distributed datasets ANOVA Kruskal-Wallis test followed by a Benjamini-Hochberg post hoc test (q = 0.05) was used for multiple datasets. When multiple parameters were compared (genotypes and treatments) a two-way ANOVA was used, followed by a post hoc Tukey test for multiple comparison correction. Significance levels are defined as p < 0.0001, p < 0.01, p < 0.05 and ns, not significant. 'n' in the legends indicates the number of animals used and analyzed. In the SING and seizure assay 'n' indicates the number of times the experiment was performed with groups of animals. The association between genotype subgroups (A1–B2) and small molecule treatment (Q10 vs R55) was tested in R (version 3.6.3). A contingency table was generated, and significance was assessed with Fisher's exact test, appropriate for small sample sizes. Data are plotted as mean ± SD or median and IQR. Specifics on the statistical test used for analysis are reported in the figure legends.

For manuscripts utilizing custom algorithms or software that are central to the research but not yet described in published literature, software must be made available to editors and reviewers. We strongly encourage code deposition in a community repository (e.g. GitHub). See the Nature Portfolio guidelines for submitting code & software for further information.

Data

Policy information about availability of data

All manuscripts must include a data availability statement. This statement should provide the following information, where applicable:

- Accession codes, unique identifiers, or web links for publicly available datasets
- A description of any restrictions on data availability
- For clinical datasets or third party data, please ensure that the statement adheres to our policy

The data generated in this study are provided in the Source Data file with this paper. Further data are available upon request from the corresponding author.

Research involving human participants, their data, or biological material

Policy information about studies with human participants or human data. See also policy information about sex, gender (identity/presentation), and sexual orientation and race, ethnicity and racism.

Reporting on sex and gender	not applicable
Reporting on race, ethnicity, or other socially relevant groupings	not applicable
Population characteristics	not applicable
Recruitment	not applicable
Ethics oversight	not applicable

Note that full information on the approval of the study protocol must also be provided in the manuscript.

Field-specific reporting

Please select the one below that is the best fit for your research. If you are not sure, read the appropriate sections before making your selection.

- [x] Life sciences
- [ ] Behavioural & social sciences
- [ ] Ecological, evolutionary & environmental sciences

For a reference copy of the document with all sections, see nature.com/documents/nr-reporting-summary-flat.pdf

Life sciences study design

All studies must disclose on these points even when the disclosure is negative.

Sample size	No statistical methods were used to predetermine sample size. Sample size was chosen based upon previous studies in the field, the technical difficulty and throughput of the assay. The sample size (n) of each experiment is indicated in the figure legends in the main manuscript and supplementary information files. Sample sizes for experiments were consistent with those commonly used in the field, typically ranging from 5 to 20 brains, ≥5 groups of flies for SING assay, ≥5 flies for ERG assay and ≥69 flies for behavioral ethoscope recordings.
Data exclusions	No data points were excluded from analysis.
Replication	Each experiment was replicated two or more times, unless otherwise indicated.
Randomization	Animals were selected based on their genotype and then randomly assigned for use in experiments.
Blinding	No blinding was performed in this study, and techniques were applied uniformly to all samples in parallel.

Reporting for specific materials, systems and methods

We require information from authors about some types of materials, experimental systems and methods used in many studies. Here, indicate whether each material, system or method listed is relevant to your study. If you are not sure if a list item applies to your research, read the appropriate section before selecting a response.

Materials & experimental systems

n/a	Involved in the study
☐	☒ Antibodies
☒	☐ Eukaryotic cell lines
☒	☐ Palaeontology and archaeology
☐	☒ Animals and other organisms
☒	☐ Clinical data
☒	☐ Dual use research of concern
☒	☐ Plants

Methods

n/a	Involved in the study
☒	☐ ChIP-seq
☒	☐ Flow cytometry
☒	☐ MRI-based neuroimaging

Antibodies

Antibodies used	Rabbit polyclonal anti-TH Millipore Cat# AB152, RRID: AB_390204<br>Mouse monoclonal anti-DLG DSHB DSHB Cat# 4F3 anti-discs large;<br>RRID: AB_528203<br>Goat anti-Rabbit IgG Alexa Fluor™ 488 Life Technologies Cat# A-11034<br>RRID: AB_2576217<br>Goat anti-Mouse IgG Alexa Fluor™ 555 Life Technologies Cat# A-21424<br>RRID: AB_141780
Validation	Rabbit polyclonal anti-TH Millipore Cat# AB152: stated by Millipore: Affinity Purfied, Quality Assurance: Routinely evaluated by Western Blot on PC12 lysates.<br>Mouse monoclonal anti-DLG DSHB Cat# 4F3 anti-discs large: it is an antibody commonly used in D. melanogaster research e.g. Wu et al. 2011 https://doi.org/10.1016/j.cub.2011.02.041

Animals and other research organisms

Policy information about studies involving animals; ARRIVE guidelines recommended for reporting animal research, and Sex and Gender in Research

Laboratory animals	Drosophila melanogaster (all strains are listed in the method section supplementary table 3) at 6±1 d, 26±2 d and 42±2 d unless otherwise indicated. All fly lines were generated in this study except for:<br>w[1118]; gmRPL11/CyO Tb[1] FlyBase ID: FBal0230528 a gift from Nicolas Tapon<br>w[*]; Df(3R)Pdf[attP], TI{RFP[3xP3.cUa]=TI}Pdf[attP] BDSC_84561<br>w[*]; P{w[+mC]=EP}park[1]/TM3, Sb[1] Ser[1] BDSC_34747<br>w[*]; park[Delta21]/TM3, P{w[+mC]=GAL4-Kr.C}DC2, P{w[+mC]=UAS-GFP.S65T}DC10, Sb[1] BDSC_ 51652
Wild animals	The study did not involve wild animals.
Reporting on sex	Male flies were used throughout the study as the incidence of Parkinson's disease (PD) and Parkinsonism is significantly higher in males than in females with an overall 1.5 times higher risk (Wooten et al., 2004). Moreover, disease progression seems to differ in males and females (Haaxma et al., 2006). The underlying reasons for this remain unknown. In addition to lifestyle differences, such as exposure to toxic substances, a contribution of sex related differences, such as hormonal levels, are highly likely. The contribution of ethnicity to PD risk in contrast to geographic location is not fully understood (Ben-Joseph et al., 2020).
Field-collected samples	The study did not involve field collected samples.
Ethics oversight	Ethical approval is not required for Drosophila research in the EU.

Note that full information on the approval of the study protocol must also be provided in the manuscript.

Plants

Seed stocks	not applicable
Novel plant genotypes	not applicable
Authentication	not applicable
